# Identification, evolution and expression analyses of the whole genome-wide *PEBP* gene family in *Brassica napus* L.

**DOI:** 10.1186/s12863-023-01127-4

**Published:** 2023-05-03

**Authors:** Yanling Li, Lu Xiao, Zhi Zhao, Hongping Zhao, Dezhi Du

**Affiliations:** 1 Academy of Agricultural and Forestry Sciences, Qinghai University, Xining, 810016 China; 2 Laboratory for Research and Utilization of Qinghai Tibet Plateau Germplasm Resources, Xining, 810016 China; 3 The Qinghai Research Branch of the National Oil Crop Genetic Improvement Center, Xining, 810016 China; 4 Key Laboratory of Spring Rapeseed Genetic Improvement of Qinghai Province, Xining, 810016 China; 5 Qinghai Spring Rape Engineering Research Center, Xining, 810016 China; 6 Spring Rape Scientific Observation Experimental Station of Ministry of Agriculture and Rural Areas, Xining, 810016 China

**Keywords:** *B.napus*, *PEBP*, Evolutionary analysis, Gene duplication, Expression analysis

## Abstract

**Background:**

With the release of genomic data for *B.rapa*, *B.oleracea,* and *B.napus*, research on the genetic and molecular functions of *Brassica* spp. has entered a new stage. *PEBP* genes in plants play an important role in the transition to flowering as well as seed development and germination. Molecular evolutionary and functional analyses of the *PEBP* gene family in *B.napus* based on molecular biology methods can provide a theoretical basis for subsequent investigations of related regulators.

**Results:**

In this paper**,** we identified a total of 29 *PEBP* genes from *B.napus* that were located on 14 chromosomes and 3 random locations. Most members contained 4 exons and 3 introns; motif 1 and motif 2 were the characteristic motifs of *PEBP* members. On the basis of intraspecific and interspecific collinearity analyses, it is speculated that fragment replication and genomic replication are the main drivers of for the amplification and evolution of the *PEBP* gene in the *B.napus* genome. The results of promoter *cis*-elements prediction suggest that *BnPEBP* family genes are inducible promoters, which may directly or indirectly participate in multiple regulatory pathways of plant growth cycle. Furthermore, the tissue-specific expression results show that the expression levels of *BnPEBP* family genes in different tissues were quite different, but the gene expression organization and patterns of the same subgroup were basically the same. qRT***‒***PCR revealed certain spatiotemporal patterns in the expression of the *PEBP* subgroups in roots, stems, leaves, buds, and siliques, was tissue-specific, and related to function.

**Conclusions:**

A systematic comparative analysis of the *B.napus PEBP* gene family was carried out at here. The results of gene identification, phylogenetic tree construction, structural analysis, gene duplication analysis, prediction of promoter *cis*-elements and interacting proteins, and expression analysis provide a reference for exploring the molecular mechanisms of *BnPEBP* family genes in future research.

**Supplementary Information:**

The online version contains supplementary material available at 10.1186/s12863-023-01127-4.

## Background

As proteins with conserved functions throughout evolution, the phosphatidylethanolamine-binding protein (PEBP) family are widespread among archaeans, prokaryotes, and eukaryotes [1, 2]. In animals, as RAF kinase inhibitors, PEBP family members regulate the cell cycle and growth [3, 4]. In plants, the PEBP family has been shown to play a crucial role in regulating growth and morphogenesis. Phosphatidylethanolamine is a major component of plant biofilms and is responsible for protein recognition and signal transduction in signal transmission [5]. Studies have shown that the *PEBP* gene family has been conserved during evolution. The proteins encoded by family members contain a single and highly conserved PEBP/RKIP domain, which constitutes 80% of the gene coding sequence [6, 7]. *PEBP* genes in plants were originally cloned from inflorescence mutants, including the snapdragon *CENTRORADIALIS* (*CEN*) gene [8], the *TERMINAL FLOWER 1 (TFL1)* gene in *A.thaliana* [5], and the *SELF-PRUNING* (*SP*) gene in tomato [9]. It is believed that *PEBP* family genes are generally key regulators of the transition from vegetative growth to reproductive growth and determine the morphological structure of plants [8, 10, 11].

Currently, the *PEBP* gene family in plants is divided into three subfamilies, namely, *FLOWERING LOCUS T* (*FT*)*-like* proteins, *MOTHER OF FT AND TFL1* (*MFT*)*-like* proteins, and *TERMINAL FLOWER 1* (*TFL1*)*-like* proteins. Among them, the *FT-like* subfamily has two members, *FLOWERING LOCUS T* (*FT*) and *TWIN SISTER FT* (*TSF*); the *MFT-like* subfamily has only one member, the *MFT* gene; and the *TFL1-like* subfamily has three members: *BROTHER OF FT* and *TFL1* (*BFT*), *TERMINAL FLOWER 1* (*TFL1*), and *A.thaliana CENTRORADIALIS* (*ATC*) [12, 13].

The *FT* gene and its homologues have been identified and cloned in a variety of plant species. In the photoperiod pathway of flowering, long days can induce the expression of *FT*, and in the vernalization pathway, long-term light induction can increase the instantaneous accumulation of *FT* mRNA and promote the flowering of vernalized material [14–16]. *TSF*, as a member of the *FT-like* subgroup, performs the same function as *FT*. *TSF* has been shown to be a flowering integration factor and to promote flowering under short-day conditions by inducing cytokines [17, 18]. The main functions of *TFL* are to maintain vegetative growth and to promote indeterminate inflorescence growth. There are two *TFL1-like* homologous genes in pea: *DETERMINATE* (*DET*) and *LATE FLOWERING* (*LF*). *DET* maintains infinite inflorescence growth and is expressed in the apical meristem after the completion of the floral transition. When *DET* is mutated, the apex changes from infinite growth to limited growth, but the flowering time does not change, which is similar to the effects of the *CEN* gene in snapdragon, where *LF* induces flower formation. Both shoot apical meristem sites and shoot tips in the vegetative growth stage express *CEN*. When *LF* is mutated, flowering is advanced, but the state of the apical meristem does not change [19]. In *A.thaliana*, *TFL1* controls plant morphological structure by regulating the *LEAFY* (*LFY*) and *APETALA 1 (AP1*) genes in the apical meristem. In wild-type *A.thaliana*, the *LFY* and *AP1* genes are upregulated at the top of the floral meristem, thus producing a flower at the top of the stem and transforming the indeterminate inflorescence into a determinate inflorescence, which delays the flowering of the *tfl1* mutant [5, 20]. The *ATC* gene in *A.thaliana* generates flower buds in a non-cell-autonomous manner. The *ATC* gene is specifically expressed in vascular tissue to produce ATC proteins. *ATC* proteins are then transported from vascular tissue to the shoot apex and bind to the *FD* protein to inhibit the expression of *AP1*, thus delaying flowering [21, 22]. *RAS-RELATED GTP-BINDING NUCLEAR PROTEIN 2* (*RAN2)* is the homologous gene of *ATC* in rice. Overexpression of *RAN2* in *A.thaliana* delays flowering and increases the number of branches. Overexpression of *RAN2* in rice prolongs vegetative growth and increases branching [23]. Studies have shown that overexpression of *BFT* in *RAN2 A.thaliana* delays flowering and produces inflorescence structures similar to those produced in response to overexpression of the *TFL1* gene. Thus, it is speculated that the *BFT* and *TFL1* genes have similar functions [24]. In addition, under high-salt stress, BFT protein and FT protein compete for binding of the transcription factor *FD* in the nucleus, and interference with the binding of *FT-FD* also leads to delayed flowering [25, 26]. A previous study found that after the *MFT* gene was overexpressed in *A.thaliana*, the flowering time decreased slightly, but the response was not as significant as that to overexpression of the *FT* gene [27]. A study of *A.thaliana MFT* showed that *MFT* was specifically expressed in seeds in response to abscisic acid (ABA) and gibberellic acid (GA) signaling pathways and participated in the regulation of seed germination [28]. The wheat *MFT* homologue plays a key role in regulating seed germination during wheat seed development and regulating the effect of temperature on seed dormancy [29].

*B.napus (Brassica napus,* AACC, 2n = 38*)*, the largest oil crop species in China, belongs to the cruciferous family, to the same as *A.thaliana.* The growth cycle and flowering time of plants directly determine their ecological adaptability, and studies have confirmed that the flowering time and maturity of *B.napus* are significantly positively correlated [30]. In recent years, in some areas of southern China with the "rape-rice-rice" farming method of three crops a year, the contradiction between *B.napus* and rice stubble has continued to intensify, resulting in a large amount of farmland being idle in the winter and the area of rapeseed planting continuing to decrease. In northwestern China, *B.napus* does not mature properly due to the high altitude and low temperature. The key factor to solve these problems is using heterosis to cultivate early-maturing *B.napus* varieties [31, 32]. The molecular mechanisms regulating the growth and development of *A.thaliana* have been studied. By means of map-based cloning, some quantitative trait loci (QTLs) and genes related to growth and development were successively obtained in *B.napus.* It has been demonstrated that members of the *PEBP* gene family play an important role in the growth and development of soybean, cotton, wheat, and other crop species [33]. However, studies on the identification, evolution, and expression of the *PEBP* gene family in *B.napus* are limited. In this study, the members of the *A.thaliana PEBP* gene family were used as reference sequences, and the BLASTP method was used to determine the *PEBP* gene family members in *B.napus* obtain information on this family in the ancestor species *B.rapa* and *B.oleracea,* construct a phylogenetic tree, and analyse gene structure. Analysis of protein physicochemical properties, selection pressure, predicted promoter *cis-*elements and interacting proteins, and tissue-spiecific expression can provide a theoretical reference for the study of candidate genes for flowering traits in *B.napus*.

## Results

### Identification of *PEBP* family members

To screen and identify members of the *PEBP* gene family in *B.napus*, *B.rapa,* and *B.oleracea*, six *PEBP* protein sequences from *A.thaliana* were used as query sequences for BLASTP analysis via the BRAD website. Ultimately, 14, 14, and 29 *PEBP* gene family members were identified in *B.rapa*, *B.oleracea,* and *B.napus*, respectively. Among the 29 *BnPEBP* genes identified, *BnaC03G0275900ZS* was the longest (over 3724 bp), and *BnaA04G0179000ZS* was the shortest (only 445 bp). The three subgroups *FT-like*, *MFT-like,* and *TFL-like* of the *PEBP* gene family in *B.napus* contain 8, 4, and 17 members, respectively. *FT* has six homologous genes, *TSF* has two homologous genes, *MFT* has four homologous genes, *TFL1* has six homologous genes, *ATC* has nine homologous genes, and *BFT* has two homologous genes. The data in Table 1 suggest that the *A.thaliana AT1G65480* and *AT2G27550* genes have no homologues in *B.rapa* and *B.oleracea*. However, there was 1 *BnaFT-C04* homologue and 3 genes (*Bnascaffold0139G0000200ZS*, *Bnascaffold0105G0000300ZS*, and *Bnascaffold0027G0054400ZS*) homologues in *B.napus*, and it is preliminarily speculated that the genes formed in *B.napus* through the distant hybridization of *B.rapa* and *B.oleracea*. Genome doubling leads to gene expansion, which can persist after tens of thousands of years of evolution in *B.napus,* indicating that these genes have an important impact on the growth and development of this species. In addition, *BolTFL-C04*, *BraATC-A04*, and *BraATC-Ann* have no homologues in *B.napus*. There are two reasons for this speculation. First, these three genes may have been eliminated during the genome evolution of *B.rapa* and *B.oleracea*; second, they may have been pressnt in *B.rapa* and *B.oleracea* during hybridization but doubled in *B.napus*. It is possible that some functionally similar genes replaced the redundant *PEBP* genes. In general, although the *PEBP* gene family has been lost during the evolution of *B.napus*, the *FT-like* and *ATC-like* genes in the *B.napus* genome have been amplified to a certain extent, which means that gene replication plays a very important role in the evolution of *B.napus*.

**Table 1:** Identification of *PEBP* family genes from *A.thaliana*, *B. rapa*, *B.oleracea*, and *B.napus*

*A. thaliana*	*B. rapa* homologues	*B. oleracea* homologues	*B. napus* homologues	Chr	Gene start	Gene end	Gene length
*AT1G65480*	*BraA02g016700.3C*	*-*	*BnaA02G0156900ZS*	A02	9,104,462	9,107,270	2808
*AT1G65480*	*BraA07g040390.3C*	*-*	*BnaA07G0282700ZS*	A07	26,253,195	26,255,955	2760
*AT1G65480*	*BraA07g031650.3C*		*BnaA07G0365100ZS*	A07	30,986,418	30,988,169	1751
*AT1G65480*	*-*	*BolC02g021840.2J*	*BnaC02G0200600ZS*	C02	16,837,203	16,838,959	1756
*AT1G65480*	*-*	*-*	*BnaC04G0181400ZS*	C04	17,070,818	17,073,769	2951
*AT1G65480*	*-*	*BolC06g036950.2J*	*BnaC06G0323800ZS*	C06	42,937,446	42,939,746	2300
*AT4G20370*	*-*	*BolC02g033320.2J*	*BnaC02G0302200ZS*	C02	29,002,223	29,003,948	1725
*AT4G20370*	*-*	*BolC04g020210.2J*	*-*	-	-	-	
*AT4G20370*	*-*	*BolC06g048270.2J*	*BnaC06G0428800ZS*	C06	50,699,507	50,700,480	973
*AT1G18100*	*BraA06g013820.3C*	*-*	*BnaA06G0123900ZS*	A06	7,315,661	7,317,429	1768
*AT1G18100*	*BraA09g057390.3C*	*-*	*BnaA09G0615100ZS*	A09	60,355,245	60,356,970	1725
*AT1G18100*	*-*	*BolC05g015780.2J*	*BnaC05G0152000ZS*	C05	9,745,846	9,747,465	1619
*AT1G18100*	*-*	*BolC08g051080.2J*	*BnaC08G0470600ZS*	C08	49,930,224	49,931,954	1730
*AT1G18100*	*BraA02g001100.3C*	*-*	*BnaA02G0014100ZS*	A02	1,037,220	1,038,340	1120
*AT5G03840*	*BraA03g001350.3C*	*-*	*BnaA03G0012400ZS*	A03	588,562	589,627	1065
*AT5G03840*	*BraA10g032420.3C*	*-*	*BnaA10G0288700ZS*	A10	25,924,219	25,925,284	1065
*AT5G03840*	*-*	*BolC02g001580.2J*	*BnaC02G0013900ZS*	C02	1,087,793	1,088,870	1077
*AT5G03840*	*-*	*BolC03g001610.2J*	*BnaC03G0016500ZS*	C03	785,008	786,061	1053
*AT5G03840*	*-*	*BolC09g068190.2J*	*BnaC09G0608000ZS*	C09	67,291,091	67,292,162	1071
*AT2G27550*	*BraA03g025050.3C*	*-*	*BnaA03G0233400ZS*	A03	12,179,465	12,181,287	1822
*AT2G27550*	*BraA04g019800.3C*	*-*	*BnaA04G0179000ZS*	A04	18,389,029	18,389,474	445
*AT2G27550*	*BraA04g019830.3C*	*-*	*-*	-	-	-	
*AT2G27550*	*BraA07g018240.3C*	*-*	*BnaA07G0155500ZS*	A07	18,656,500	18,658,099	1599
*AT2G27550*	*BraAnng000070.3C*	*-*	*-*	-	-	-	
*AT2G27550*	*-*	*BolC03g029060.2J*	*BnaC03G0275900ZS*	C03	17,403,007	17,406,731	3724
*AT2G27550*	*-*	*BolC04g054340.2J*	*BnaC04G0205900ZS*	C04	20,146,840	20,149,549	2709
*AT2G27550*	*-*	*BolC04g022880.2J*	*BnaC04G0478300ZS*	C04	60,565,511	60,567,293	1782
*AT2G27550*	*-*	*-*	*Bnascaffold0139G0000200ZS*	scaffold0139	13,676	15,271	1595
*AT2G27550*	*-*	*-*	*Bnascaffold0105G0000300ZS*	scaffold0105	15,295	16,889	1594
*AT2G27550*	*-*	*-*	*Bnascaffold0027G0054400ZS*	scaffold0027	4,590,310	4,591,903	1593
*AT5G62040*	*-*	*BolC03g059420.2J*	*BnaC03G0559000ZS*	C03	42,600,265	42,601,116	851
*AT5G62040*	*BraA06g025510.3C*	*-*	*BnaA06G0273500ZS*	A06	37,354,175	37,355,019	844

### Phylogenetic analysis and classification of *PEBP* genes

**Fig. 1:**
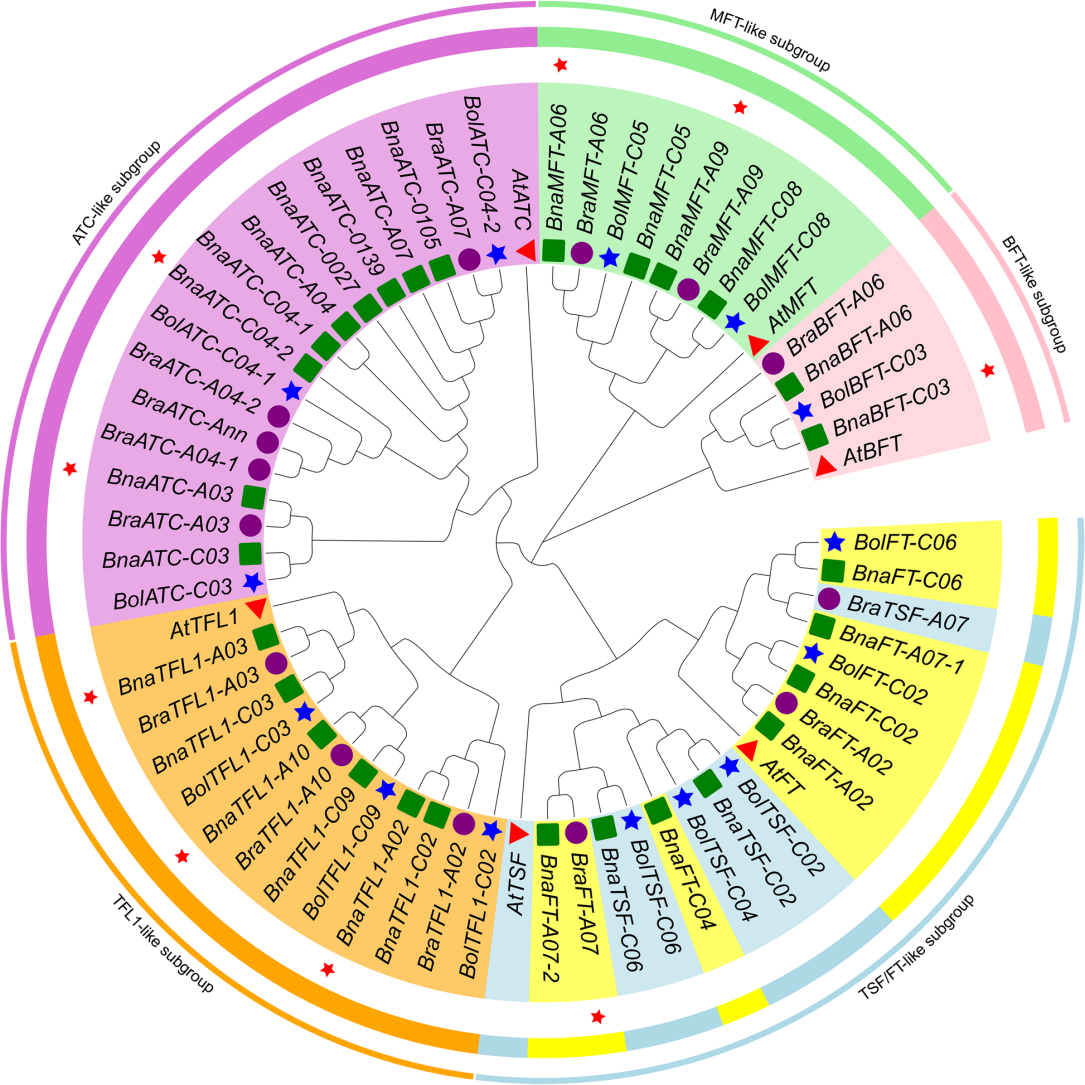
Phylogeny of the PEBP family in *B.napus* *,* *B.rapa*, *B.oleracea*, and *A.thaliana*. Note: The proteins are clustered into six subgroups. Yellow, light blue, orchid, light green, orange and pink sections indicate the subgroups of *PEBP* proteins. *BnaPEBPs* are marked with green dots, *BraPEBPs* are marked with purple dots, *BolPEBPs* are marked with blue stars, *AtPEBPs* are marked with red triangles, and 9 *BnPEBPs* core genes are marked with red stars

To explore the phylogenetic relationships of the *PEBP* gene family in cruciferous species, we investigated a total of 63 *PEBP* members from *B.napus* (29 genes), *B.rapa* (14 genes), *B.oleracea* (14 genes), and *A.thaliana* (6 genes). Sequence alignment was performed using Jalview software, and a phylogenetic tree was constructed by the neighbour-joining method using MEGA software (Fig. 1). The phylogenetic tree divided these 63 proteins into five subgroups: the *ATC-like* subgroup, the *TFL1-like* subgroup, the *TSF/FT-like* subgroup, the *BFT-like* subgroup, and the *MFT-like* subgroup. Among them, the *ATC-like* subgroup and *TSF/FT-like* subgroup had the most members at 18 each, while the *BFT-like* subgroup had the fewest members at 5 (Fig. 1). According to the clustering in Fig. 1, the *PEBP* family members of the A sub-genome in *B.napus* may be derived from the A genome of *B.rapa*, and the corresponding members of the C sub-genome may be derived from the C genome of *B.oleracea*. These results further suggest that genome doubling is responsible for the increase in the gene copy number of *PEBP* family members in *B.napus*.

### Gene structure and motif compositions

**Fig. 2:**
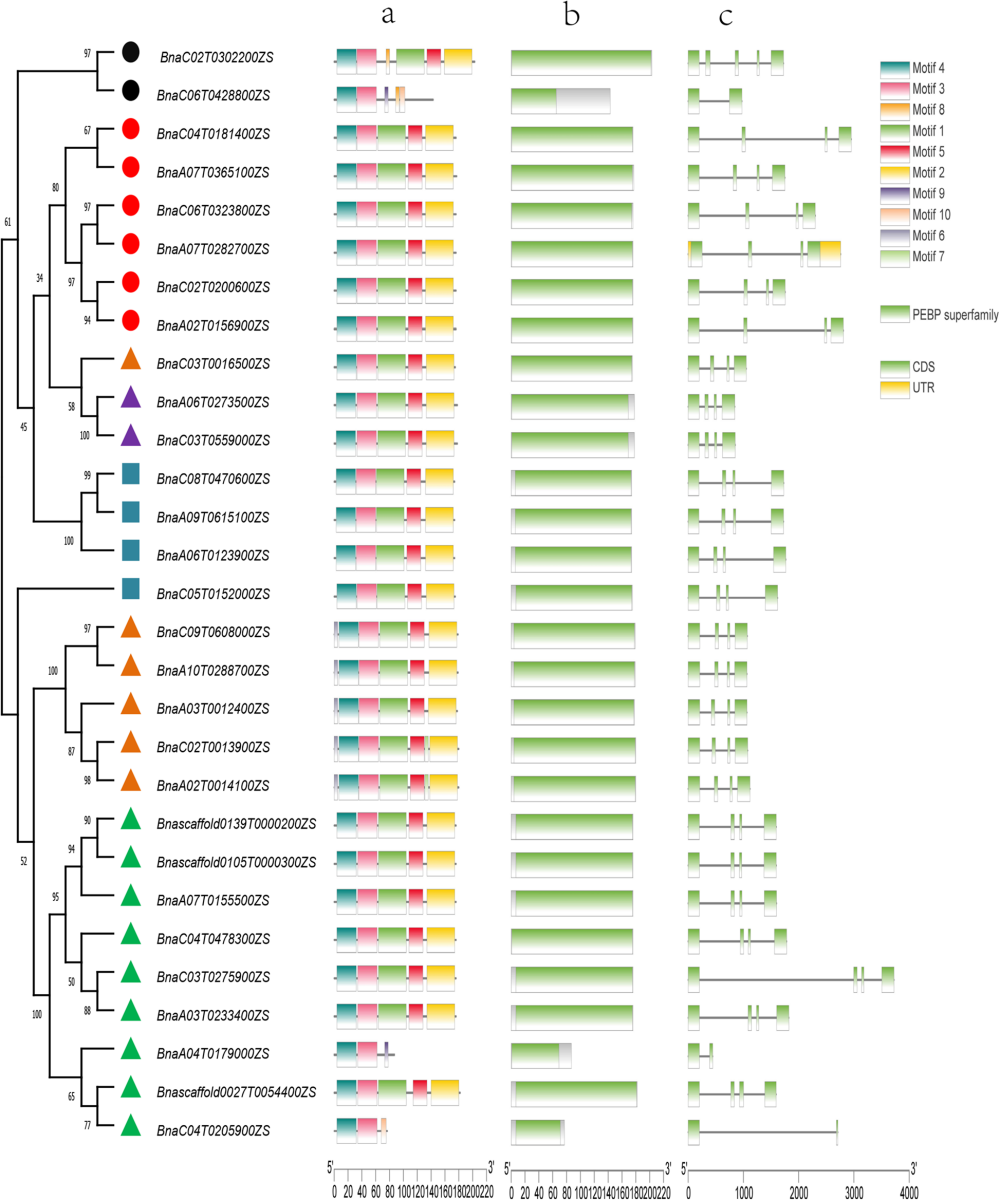
Construction of a phylogenetic tree of 29 *PEBP* genes in *B.napus* with TBtools. Note: The visual display of the *BnPEBP* evolutionary tree, gene motifs (**a**), domains (**b**) and structure (**c**)

It is known that structure determines function. To analyses the function of the *PEBP* gene family, the structure of 29 *BnPEBP* gene family members was analysed, and the results showed that the gene structure of *BnPEBP* family members was highly conserved (Fig. 2). The motif prediction results (Fig. 2a) show that motif 3, motif 4, motif 5, and motif 6 are characteristic motifs of the *BnPEBP* gene family, except for *BnTSF-C06*, *BnATC-A04,* and *BnBFT-C04,* which contain only motif one and motif two. These results suggest that *BnPEBP* members from different subgroups have a simple structure and that specific motifs may lead to different roles for these genes based on the function of the protein produced. Figure 2b shows that the *BnPEBP* gene family has the same conserved domain. Gene structure analysis (Fig. 2c) showed that, except for *BnTSF-C06*, *BnATC-A04*, and *BnBFT-C04*, which contain 2 exons and 1 intron, most members contain 4 exons and 3 introns. Combined with phylogenetic tree analysis results, these results revealed that there were also differences in terms of the unified score, such as for *BnTSF-C06* in *BnTSF* and for *BnATC-C04* and *BnATC-A04* in *BnATC*. The structures of these three genes are relatively similar, while the length of their introns is different; however, since the intron is removed during transcription and translation, the function of the gene is not affected. This suggests that transposable elements may have been inserted into these genes during evolution, resulting in the fragmentation of the coding sequence into multiple segments. In addition, the different combinations of introns and exons may be due to the different splicing methods used by genes during evolution, which can lead to proteins with specific functions being produced to meet certain biochemical requirements.

### Chromosomal distribution and homology analysis

**Fig. 3:**
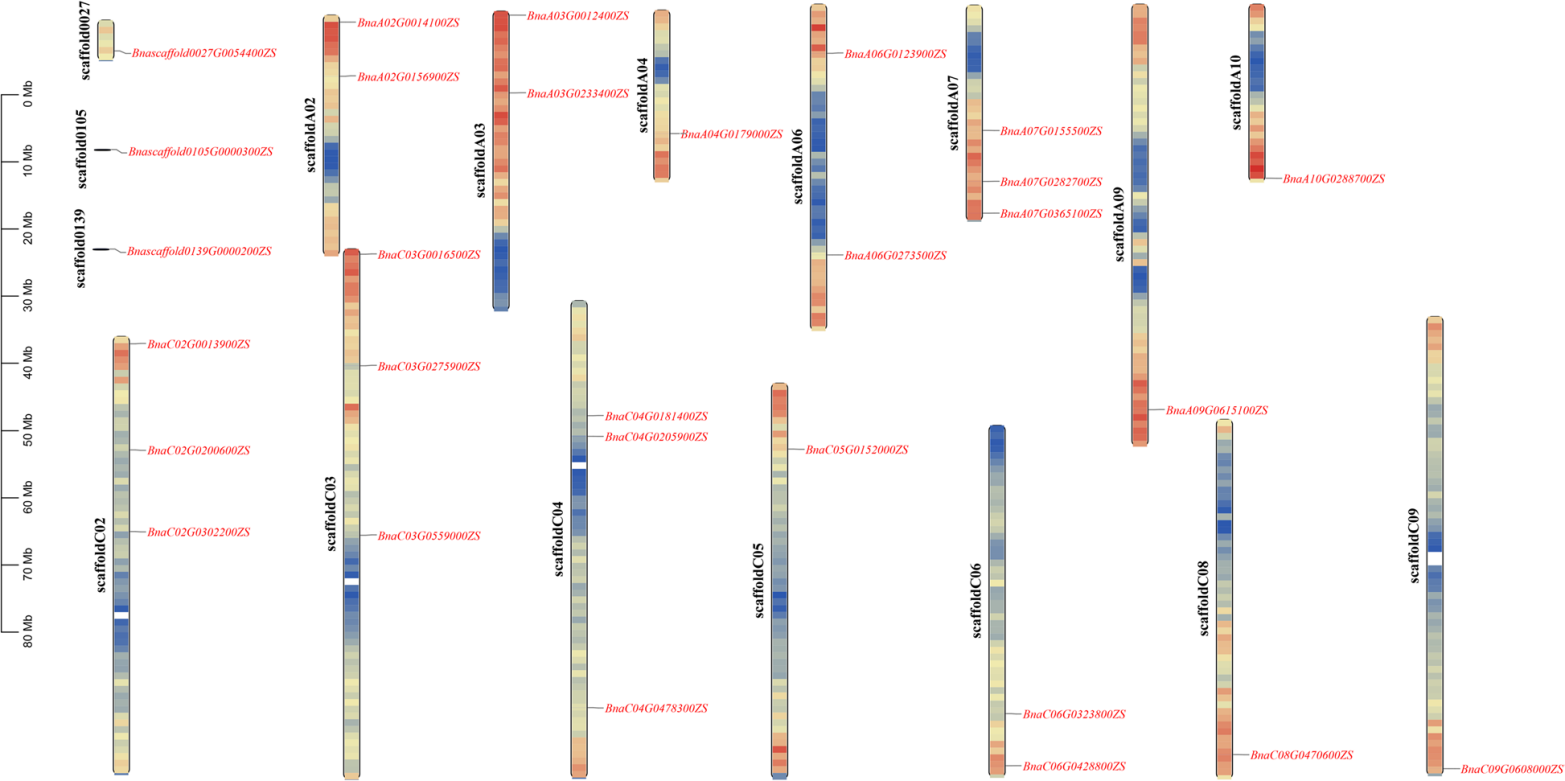
Distribution of PEBP family genes on the chromosomes of *B.napus*

Analysis of the chromosomal location of 29 members of the *PEBP* gene family in *B.napus* revealed that these genes were not distributed evenly on each chromosome. Among them, 3 genes were on unassembled scaffold segments, and the remaining 26 *BnPEBP* genes were distributed on 14 chromosomes. There were 12 *BnPEBP* genes in the A genome and 14 *BnPEBP* genes in the C genome. There were no *BnPEBP* genes on chromosomes A01, A05, and C01, and most genes were distributed on chromosomes A07, C02, C03, and C04, all of which contained 3 genes (Fig. 3).

**Fig. 4:**
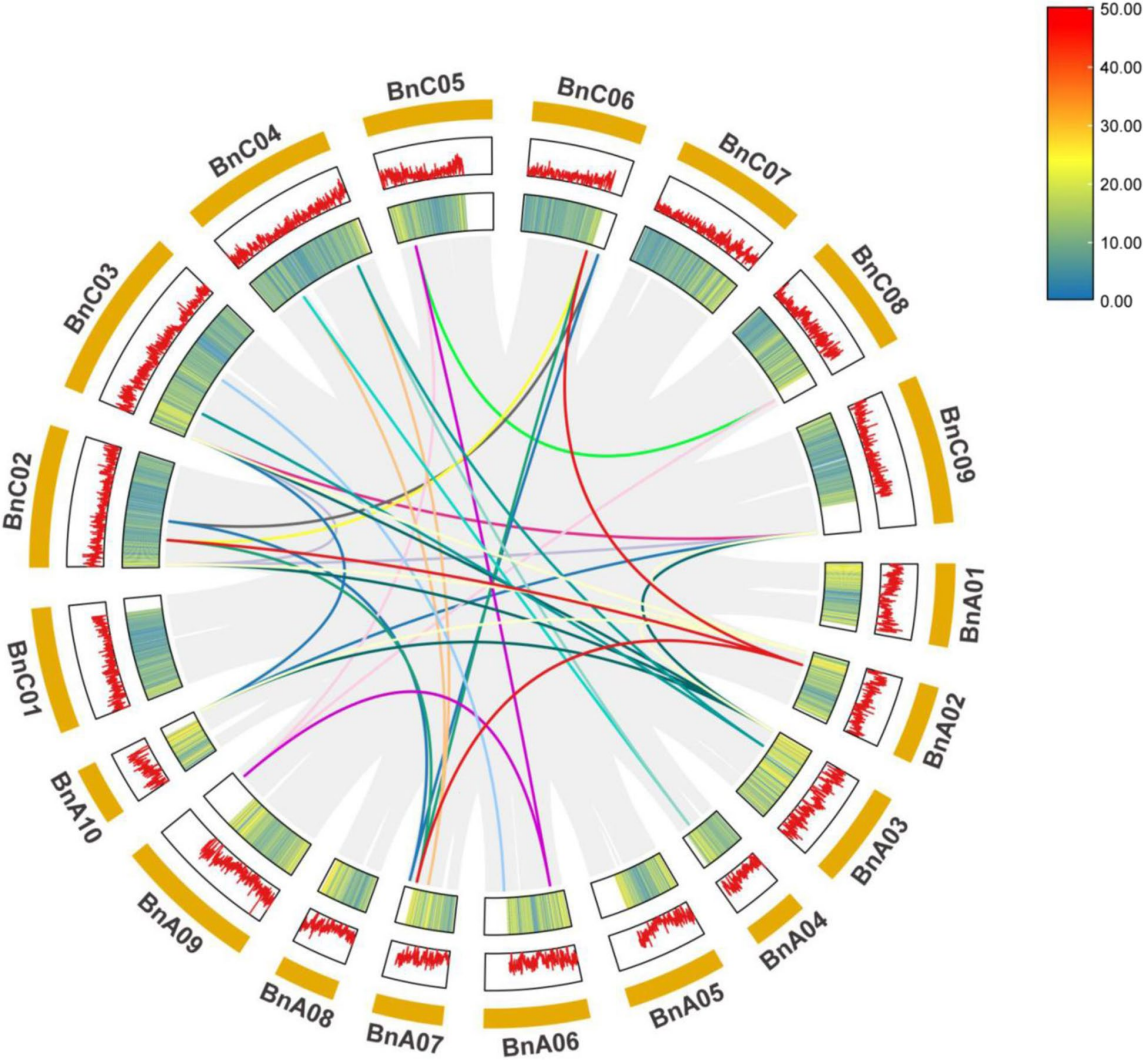
Duplication of *BnPEBP* genes on the chromosomes of *B.napus* L.

There are many duplicated genes in the *B.napus PEBP* gene family. Because the *B.napus* genome has undergone large-scale chromosomal doubling and duplication, these duplicated genes exist not only in the form of adjacent genes but also as genes on different chromosomes. In this study, to understand the potential duplication events between *BnPEBP* genes, TBtools software was used to analyse the gene duplication types of the BnPEBP family. A Circos plot was constructed (Fig. 4). The Intraspecific collinearity analysis of the *PEBP* genes in *B.napus* was carried out, and a total of 35 collinear genes were detected. Among them, there were 3, 9, and 23 pairs of homologous genes (Supplementary Table S1) between the A-A, C–C, and A-C genomes, respectively. These genes were distributed on 14 chromosomes in sub-genomes A and C of *B.napus*, and each chromosome contained one ~ six genes, and the mode of gene replication was fragment replication. In addition, some genes did not show intraspecific collinearity. Finally, we speculate that gene fragment replication may be the main mode of *PEBP* gene replication mode in the *B.napus* genome.

**Fig. 5:**
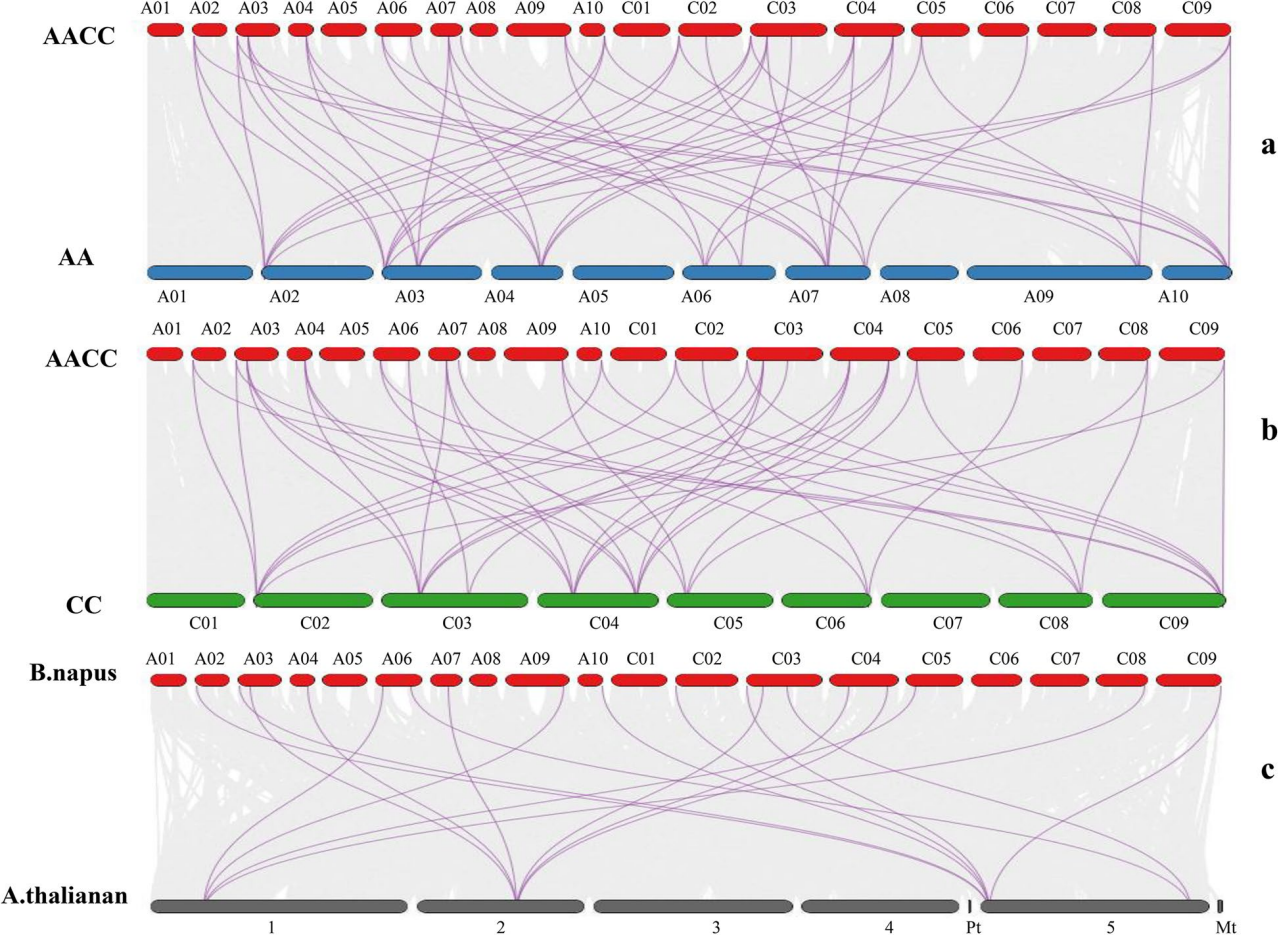
Synteny analysis of *PEBP* genes between *B.napus* and its progenitors. Note: The grey lines in the background indicate the collinear blocks within *B.napus* and its progenitor species. The purple-red lines highlight the syntenic *PEBP *gene pairs. (**a**) indicates the genome collinearity of *B.napus* (AACC) and *B.rapa *(AA), and (**b**) indicates the genome collinearity of *B.napus *(AACC) and *B.oleracea *(CC). (**c**) indicates *B.napus *(AACC) and *A. thaliana* genome collinearity. To show the completeness of the results, genes located in random regions are not shown in the figure

Based on the genome annotation information in the Ensembl Plants database, in this study, the *PEBP* genes of *A.thaliana*, *B.oleracea*, *B.rapa*, and *B.napus* were subjected to collinearity analysis and visualized by TBtools (Fig. 5). The results of the collinearity analysis showed that there were 43, 49, and 18 pairs of collinearity relationships between *BnPEBP* gene family members with *BoPEBP*, *BrPEBP*, and *AtPEBP*, respectively (Supplementary Table S2). The *BnPEBP* gene family experienced a whole-genome duplication (WGD) event during its evolution. There are 29 *B.napus PEBPs*, among which there may be at least 10 *BnPEBPs* from the *B.rapa* genome and at least 9 *BnPEBPs* from the *B.oleracea* genome. That is, genome duplication is the main reason for the rapid expansion of the *BnPEBP* gene family in *B.napus*. On the other hand, it is speculated that some members of the *PEBP* gene family formed after the duplication of the genome.

### Physicochemical properties and subcellular localization

Physicochemical analysis of *BnPEBP* family protein members showed that the number of amino acids encoded by the 29 *BnPEBP* genes varies from 76 (*BnaATC-C04*) to 202 (*BnaTSF-C02*). The molecular weight is between 8.41 and 22.96 kDa; the gene with the highest molecular weight is *BnaTSF-C02*, and the gene with the lowest is *BnaATC-C04*. Members of the BnPEBP protein family have isoelectric points (pIs) ranging from 6.38 to 9.56. The pIs of BnPEBP protein members ranged from 6.38 to 9.56. Val is the most abundant amino acid in the proteins of this family. The instability coefficient of BnPEBP protein members ranges between 26.9 and 51.58. An instability coefficient greater than 40 was used as the threshold; thus, there are 16 stable proteins in *B.napus*, while the remaining 13 proteins are unstable. The fatty acid coefficients of the 29 BnPEBP proteins range from 74.86 to 100.70, and the hydrophilicity (GRAVY) values (J) of the proteins are all less than 0, indicating that they are all hydrophilic. The subcellular localization results of 29 *BnPEBP* genes showed that 23 genes are expressed in the cytoplasm, 5 are expressed in the periplasm, and 1 gene is expressed in the extracellular space (Table S3).

### Selection pressure analysis

There may be selection pressure due to environmental stress and changes in survival during the process of species differentiation. Some genetic changes are related to the environment; that is, environmental variables can directly modify a gene, and the development of particular mutations promotes this phenomenon. However, some mutations are neutral, while others have deleterious effects, and such mutations are easily eliminated during the evolutionary process. Therefore, a mutation that is beneficial to the biological adaptation to the environment is considered to be under positive selection; however, neutral selection and purifying selection do not conducive to biological adaptations to the environment. To explore the evolutionary pressure on the BnPEBP genes, we calculated the nonsynonymous substitution rate (Ka), the synonymous substitution rate (Ks), and their ratio (Ka/Ks) between homologous gene pairs (Table S4). The Ka/Ks ratios of the *PEBP* gene family in *B.napus* were all less than 1, indicating that the evolution of the *PEBP* gene family in *B.napus* was relatively conserved and subject to strict purifying selection. The Ka/Ks ratios of the *TSF* and *FTL1* subgroups were larger than 0.2, while the Ka/Ks ratios of the *ATC*, *MFT*, *FT,* and *BFT* subgroups were smaller, indicating that the *TSF* and *TFL1* subfamilies are more conserved.

### Promoter *cis*-acting element Analysis

**Fig. 6:**
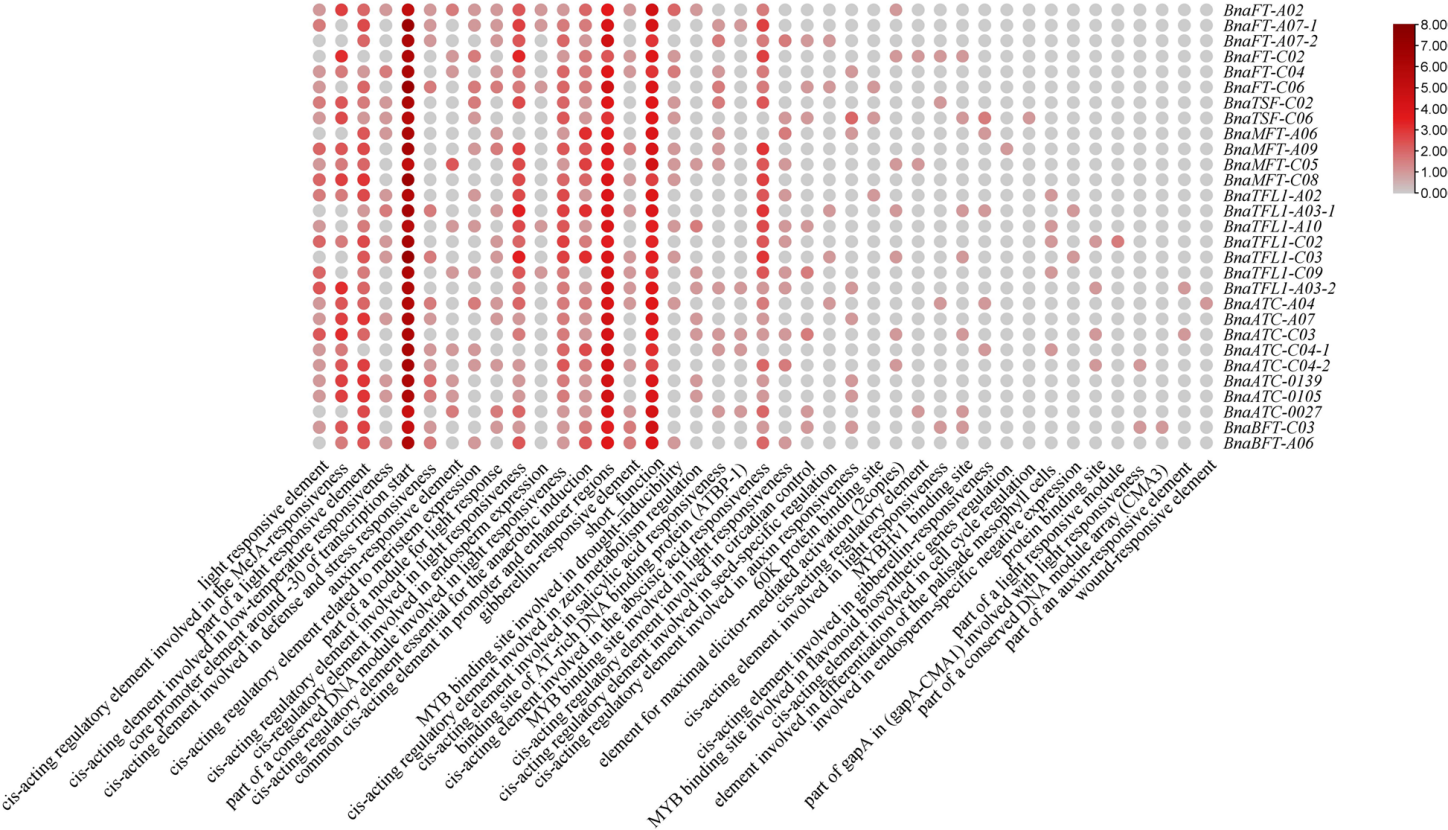
Analysis of *cis*-acting elements in the *BnPEBP* gene promoter. Notes: The image does not contain any unannotated* cis*-acting elements

*Cis-*regulatory sequences can regulate plant growth, development, and physiological metabolism by regulating gene expression. To better understand the transcriptional regulation and potential function of the *BnPEBP* genes, in this study, the promoter sequence for investigation was the 2000 bp sequence upstream of the *BnPEBP* translation start site (ATG), and the *cis-*elements in the promoter sequences were examined using the PlantCARE database. The results are shown in Fig. 6. The promoter sequences of all *BnPEBP* genes contained multiple *cis-*acting elements, indicating that *BnPEBP* genes are involved in a complex regulatory network. The analysis revealed 39 *cis-*acting elements with known functions in the *BnPEBP* gene and several *cis-*acting elements with unknown functions. *Cis-*acting elements with known functions can be categorized into five groups: the first group consists of core promoter elements, which include the TATA-box and CAAT-box; the second group consists of light-responsive elements, including the GT1 motif, the AAAC motif, SP1, the TCC motif, the GTGC motif, the TCT motif, and the AE-box; the third group consists of hormone-responsive elements, including auxin response elements (TGA elements and AUXRR-CORE elements), ABA response elements (ABREs) and gibberellin response elements (GARs); the fourth group consists of several abiotic stress response elements, including anaerobic induction elements (AREs) and defence response elements; and the fifth group consists of transcription factor-binding sites and others, including AT-rich elements and CCAAT-boxes. In addition, some elements of unknown function were also predicted. Furthermore, studies have found that some gene promoter regions contain other *cis-*regulatory elements related to plant development, including meristem expression (CAT-boxes), endosperm expression (GCN4 motifs), circadian rhythm regulation-related elements, and palisade mesophyll cell differentiation (HD-Zic1)-related *cis-*regulatory elements. Except for the core promoter element, which is the most abundant, the remaining elements are shown in Fig. 6. Several types of *cis-*elements are related to plant growth and development, which further indicates that the *PEBP* gene family regulates plant growth and development and plays an important role in this process.

### Interaction protein prediction

**Fig. 7:**
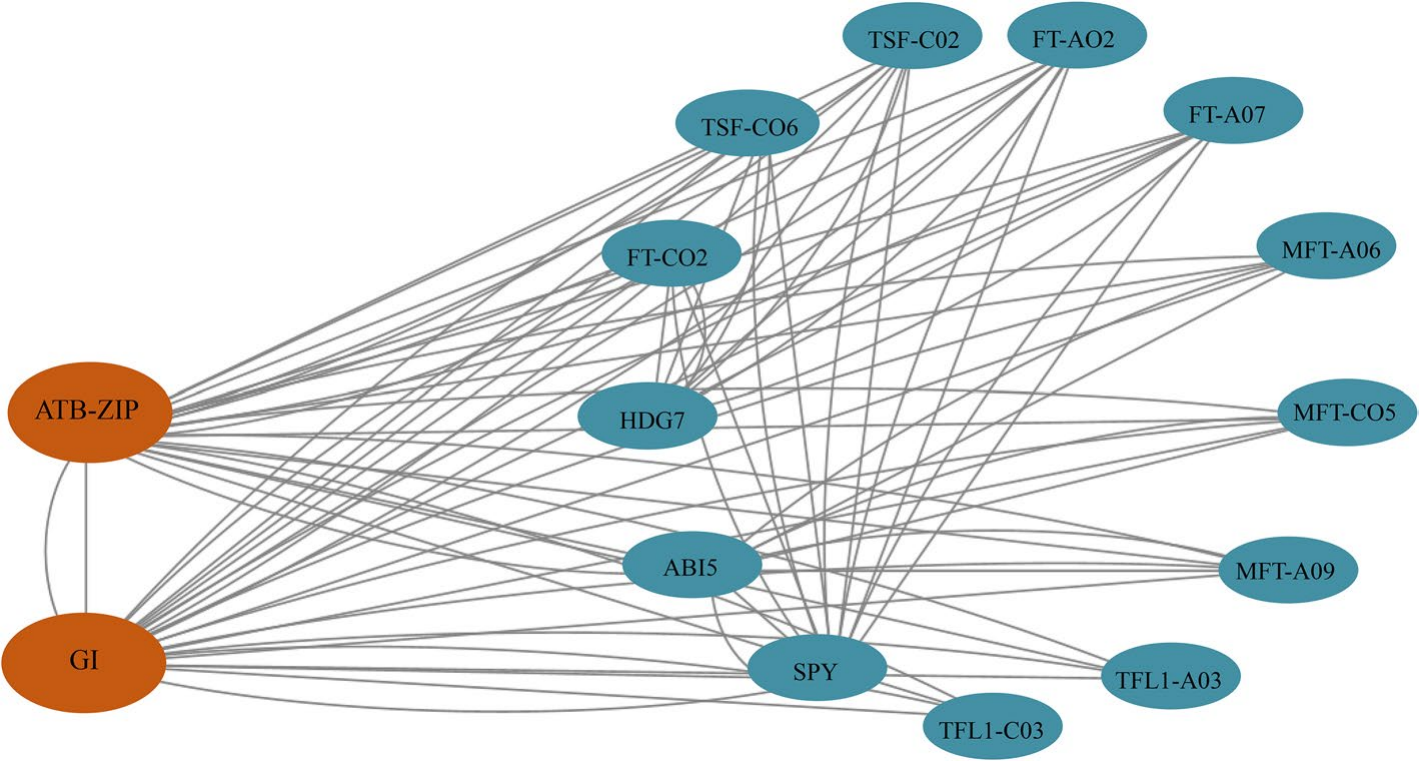
Predicted *BnPEBP* gene family protein interaction network

A protein interaction network comprising the members of the *BnPEBP* gene family identified in *B.napus* was constructed; the prediction results are shown in Fig. 7. Ten BnPEBP proteins interacted with each other and with ATBZIP, GI, SPY, ABI5, and HDG7. Five proteins were found to interact with a PPI enrichment *p* value of 1.0e-16, which means that the interactions between the proteins were stronger than those expected for a random set of proteins of the same size and degree distribution extracted from the larger set. Using the betweenness centrality index (BC) index to screen core proteins in Cytoscape software, we found that FT, TSF, MFT, and TFL1 in the BnPEBP protein family were regulated by ATBZIP and GI proteins. Various proteins in the entire protein interaction network are involved in the regulation of flowering. Among them, *ATBZIP*, a transcription factor, is involved in the regulation of plant light responses, while *GI* is a circadian rhythm gene that responds to photoperiod signals together with *CO* and acts on downstream *FT* genes to regulate flowering. As shown in Fig. 6, in addition to gene family members, SPY regulates flowering in response to the gibberellin pathway, and ABI5 is involved in ABA signaling during seed maturation and germination, including sensitivity and regulation to ABA inhibition of seed germination. The expression of some *ABA* genes is involved in the regulation of the plant growth process. HDG7 is a transcription factor that is widely involved in plant development and participates in the development of plant vascular tissue, the formation of organs, and the response to stress. Therefore, the network diagram of protein interactions indicates that BnPEBP family members directly or indirectly participate in multiple regulatory pathways during plant growth and development.

### Tissue-specific expression analysis

**Fig. 8:**
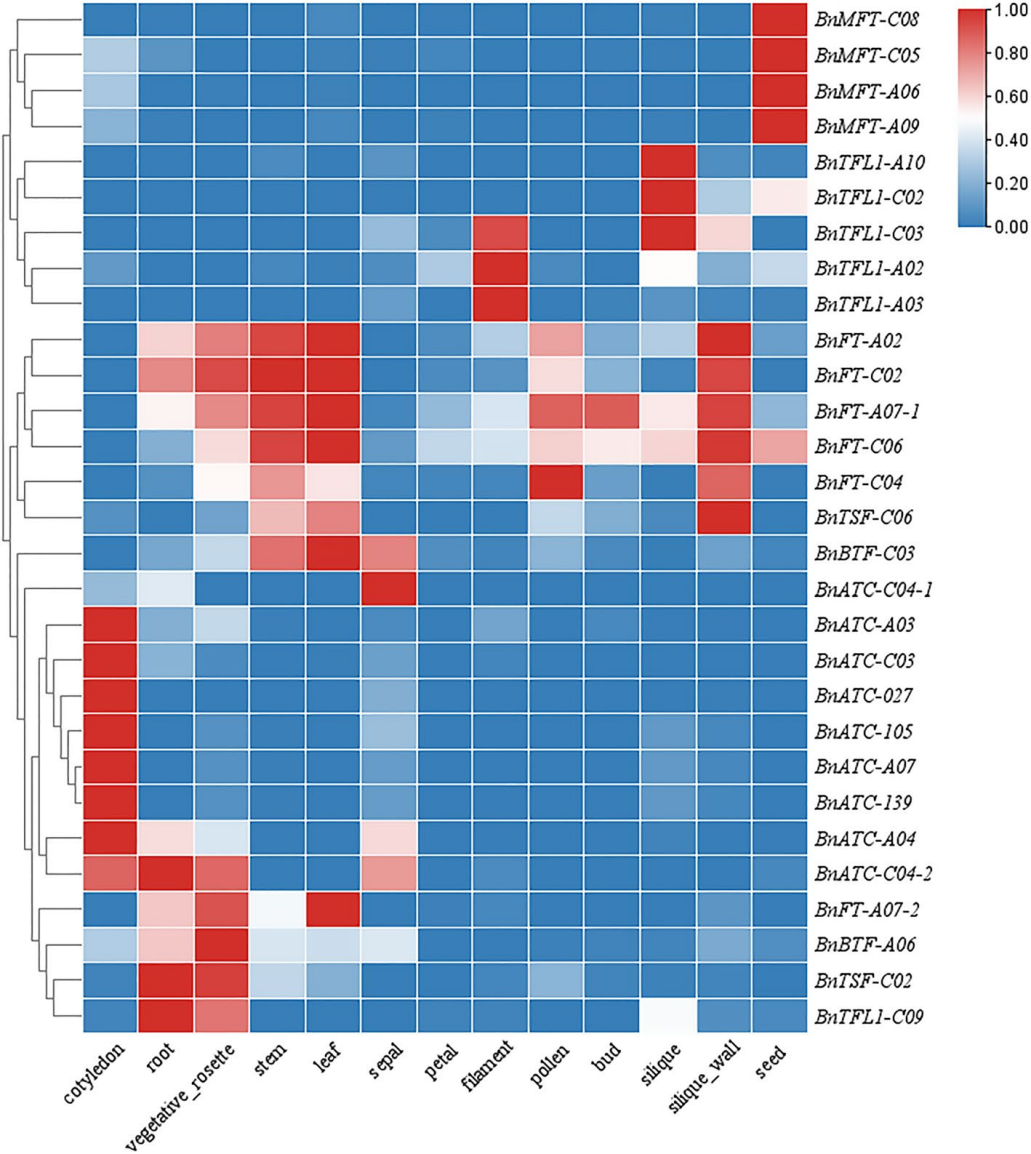
Tissue-specific expression analysis of *PEBP* gene members in* B.napus*

To understand the role of *PEBP* family genes during plant development, we used published data corresponding to different tissues of the *B.napus* cultivar "Zhongshuang 11" (cotyledon, root, vegetative rosette, stem, leaf, sepal, petal, filament, pollen, bud, silique, silique wall, and seed tissue) and performed a transcriptomic data analysis of the expression of *BnPEBP* family genes (Fig. 8). A gene expression heatmap was constructed and showed that the *BnaPEBP* family genes show tissue-specific expression in *B.napus*. The *BnaFT* gene in the *BnaFT-like* subgroup was highly expressed in the stem, leaf, and silique wall tissues, which is consistent with the production and transport of *FT* from leaves to stem apical meristems to promote plant flowering. *BnaTSF-C02*, which is typically found in the roots and during the vegetative stage, was expressed at higher levels in the rosette leaves, and *BnaTSF-C02* was expressed in the stems and leaves. The *BnaMFT-like* subgroup genes showed higher expression in the seeds than in the filaments and pollen. *BnaACT* in the *BnaTFL-like* subgroup was highly expressed in the cotyledons, but the level was low or not detected in the petal, filament, pollen, and bud tissues; *BnaTFL1* gene expression was relatively low in the early vegetative growth stage but high in the late siliques, silique walls, and seeds. The *BnaBTF* gene showed low expression across the different tissues. The above results show that the *BnPEBP* family genes in *B.napus* have different expression levels in different tissues, but the expression patterns of genes in the same subgroup are essentially the same, which may be related to their evolution from the same gene.

### Expression pattern analysis of *BnPEBP* genes by qRT‒PCR

**Fig. 9:**
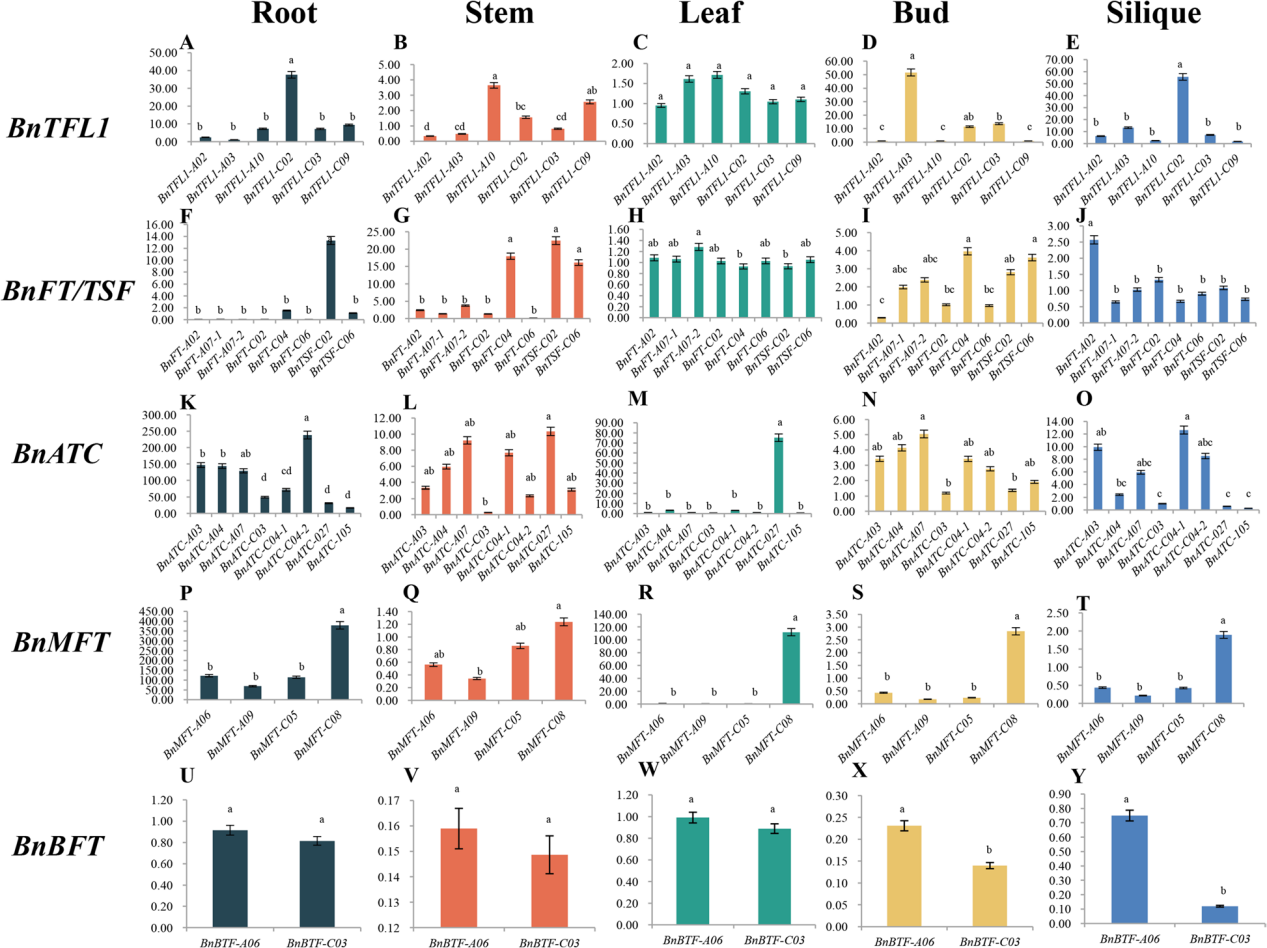
Expression levels of 5 subgroups of *PEBP* genes in roots, stems, leaves, buds, siliques of *B.napus* by qRT-PCR

The *B.napus* cultivar "Zhongshuang 9" was chosen as the experimental object based on the tissue-specific expression analysis of *BnPEBP* family genes to verify and compare the expression patterns of the same group of homologous genes in roots, stems, leaves, buds, and siliques of three plants with consistent growth at 10 days after flowering. First of all, it can be seen from Figs. 8 and 9 show that in these five tissues, the expression of genes belonging to the same subgroup was quite different. The *BnTFL1-C02* gene in the *BnTFL1-like* subgroup has a high expression level in roots and siliques, which is significantly different from the pattern in other genes, and the gene *BnTFL1-A03* has a high expression level in buds, which is significantly different from the expression pattern of other genes. Three genes in the *BnFT/TSF-like* subgroup are highly expressed in roots and stems, and most of them are expressed in leaves, buds, and siliques, but the expression level is low with little variation. The expression of the *BnATC-like* subgroup gene varies greatly among tissues, especially that of *BnATC-027,* which is high in leaves, while other genes are expressed at low or notat all. The expression level of the *BnMFT-like* subgroup gene differed significantly among the five tissues. In addition to that of *BnMFT-C08*, the expression levels of three other genes in leaves, buds, and siliques were very low, which was significantly different from that of *BnMFT-C08*; the expression level of the *BnBFT-like* subgroup was low, and there was a significant difference between the two genes in buds and siliques. Different from that in the reference genome "Zhongshuang 11,"the expression level showed an overall increase in expression in "Zhongshuang 9" It is speculated that the growth period is approximately 25 days earlier in "Zhongshuang 9" than in "Zhongshuang 11," as the *BnPEBP* family is related to plant growth and development. Therefore, there was a trend towards higher expression in the early-maturing materials. In addition, different sampling periods and different plant growth conditions are also reasons for large differences in expression patterns. Second, *BnPEBP* family genes were expressed in different tissues of *B.napus*, and the *BnaTFL1* subgroup was more highly expressed in buds than other tissues, which can be explained by the function of this gene in flowering and inflorescence growth. The *BnaMFT* subgroup and the *BnaATC* subgroup were highly expressed in the roots, which is unique. The expression of *BnaFT/TSF* in these five tissues was similar to that in the reference genome, with higher values in the aboveground tissues. The overall expression of the *BnaBFT* subgroup was low, but stable expression was observed in roots, stems, leaves, buds, and siliques.

## Discussion

*B.napus*, a naturally doubled allotetraploid crop species, has a complex genome. With the release of genomic data from *B.rapa*, *B.oleracea,* and *B.napus,* research on the genetic and molecular functions of *B.napus* has entered a new stage [34–36]. *PEBP* gene belong to a relatively small gene family in plants and play an important role in the flowering transition, seed development, and germination of plants [37]. To date, 19 *PEBP* genes have been reported in rice [38], 25 in maize [39], and 23 in soybean [40]. In this study, 14, 14, and 29 *PEBP* gene family members were identified in *B.rapa*, *B.oleracea,* and *B.napus*, respectively, by genome-wide searches. This shows that the number of *PEBP* genesdiffers among apecies, possibly related to species specificity. According to Table 1, the number of *PEBP* genes in *B.napus* is not the sum of the numbers in *B.rapa* and *B.oleracea*, indicating that members of the *PEBP* gene family were also lost or expanded with the genome duplication of *B.napus*. Phylogenetic analysis (Fig. 1) revealed that the *PEBP* genes of *B.napus* and its progenitor species, unlike those of other plant species, were divided into five subgroups, the *ATC-like* subgroup, the *TFL1-like* subgroup, the *TSF/FT-like* subgroup, the *BFT-like* subgroup, and the *MFT-like* subgroup, which were different from the three main subfamilies identified in previous research [41]. The amino acid sequences of FT and TFL1 are similar, but their functions are opposite. Hanzawa performed a detailed study on this conjecture, proving that the histidine residue (His88) at the 88th position of the TFL1 protein and the tyrosine residue (Tyr85) at the 85th position of the FT protein play a decisive role in the opposite functions [42]. The number of members of the *ATC-like* subgroup is significantly higher than that of other subgroups, indicating that this subgroup has undergone gene expansion as part of the evolutionary process and may play a more important role in the growth and development of *B.napus* than other subgroups.

Collinearity analysis can be used to predict inheritance and potential functions. The analysis of intraspecific and interspecific collinearity of the *PEBP* gene in *B.napus* shows that the mode of *PEBP* gene replication in this species is fragment replication, and the mode of gene expansion is genome doubling. There are some genes without a collinear relationship, and it is possible that chromosome recombination and homologous replacement caused by genome doubling and subsequent doubling events in *B.napus* have caused complex changes in gene expansion and loss to varying degrees. In a study of the wheat *PEBP* gene family [43], it was found that other genes also display this phenomenon.

In addition, we also predicted the *cis-*elements in the promoter; except for the core components, a large number of which are related to environmental response, such as light-responsive elements, hormone-responsive elements, and abiotic stress response elements. Combined with the function of the *PEBP* gene family in plant growth and development, it demonstrates that the promoter of *PEBP* gene family plays an important role in regulating plant growth and development. It can be inferred that the type of *PEBP* gene promoter may be an inducible promoter. In previous articles, there was little information about this; this finding can be used as a reference in the future study of gene function in this family. Furthermore, a protein interaction network comprising the members of the BnPEBP gene family identified in *B.napus* was constructed; the prediction results are shown in Fig. 7. We found that various proteins in the entire protein interaction network that are involved in the regulation of plant flowering. Among them, CO and SPY regulate plant flowering by participating in the photoperiodic and gibberellin pathways, respectively. ABI5 is involved in ABA signal transduction during seed maturation and germination, That is, the interacting proteins also prove that the PEBP protein plays an important role in the development of plant vascular tissue and the formation of organs. From this point of view, there is a certain relationship between the cis-element of the promoter and protein interaction, which can be used as an analytical direction in future research.

Tissue expression data and qRT‒PCR analysis results collected throughout the growth period revealed that BnPEBP genes were expressed at varying levels in distinct tissue regions. In this study, it was found that the *MFT-like* subgroup was specifically expressed in fruits and highly expressed in mature fruits. This result is consistent with the expression characteristics of *GmMFT* of Glycine max in fruit clips [44], suggesting that the *MFT-like* subgroup plays an important role in seed maturation. In addition, the *ATC-like* subgroupwas also highly expressed in a specific tissue: cotyledons. The *FT/TFL1-like* subgroup was expressed in multiple tissues, showing pan-tissue expression, Extensive tissue expression characteristics also indicate that *FT/TFL1-like* subgroup plays an important role in the growth and development of *B.napus.* Furthermore, we used qRT‒PCR to measure the expression levels in the roots, stems, leaves, buds, and siliques of the *B.napus* cultivar "Zhongshuang 9." The expression patterns of most *BnPEBP* homologous genes were similar and tissue specific. However, the pattern was not consistent with the tissues showing high expression in the reference cultivar "Zhongshuang 11," which may be due to the different material. In addition, different sampling periods and different plant growth conditions are also reasons for the large differences in expression patterns.

## Conclusions

In conclusion, 29 *PEBP* genes identified in *B.napus* were systematically studied in terms of gene evolution, gene structure, collinearity, *cis*-acting elements, physical and chemical properties of proteins, and gene expression patterns by bioinformatics methods. The evolution of the *PEBP* genes in *B.napus* was relatively conserved and it was proved that genome doubling and fragment duplication are the ways by which *PEBP* genes expand and replicate in *B.napus*. Prediction of *cis*-elements and analysis of interacting proteins provide new research evidence for the role of *PEBP* gene in the growth and development of *B.napus*, and the tissue expression patterns of the *PEBP* gene are closely related to their function. To date, only a few genes representing the *PEBP* gene family have been characterized in detail in *B.napus*. This study provides nearly complete information on the *BnPEBP* gene family, and our findings point to a new direction for further research into the evolution and function of *PEBP* family genes in *B.napus* and its progenitors.

## Methods

### Identification of the *PEBP* gene family members

The Stockholm model of PEBP (IPR008914) was obtained from the InterPro website (https://www.ebi.ac.uk/interpro/), and then all *A.thaliana* PEBP protein data were retrieved from the HMMER website (https://www.ebi.ac.uk/Tools/hmmer/) [45]. The CDSs of members of the *A.thaliana PEBP* gene family () were downloaded from the Arabidopsis Information Resource (TAIR) website (https://www.arabidopsis.org/). To identify *PEBP* gene candidates, the CDSs and protein sequences of the *A.thaliana PEBP* gene family members were used to search the protein database of the *B.napus* genome and its ancestral species by BLASTP (e value ≤ 1e^−^5) with the BRAD database (http://brassicadb.cn). Among them, Zhongshuang 11 was selected as the reference genome (*Brassica napus*, AACC, 2n = 38) for *B.napus*, Brara_Chiifu_V3.0 was used as the reference genome (*Brassica rapa*, AA, 2n = 20) for *B.rapa*, and Braol_JZS_V2.0 was used for the *B.oleracea* reference genome (*Brassica oleracea*, CC, 2n = 18).

### Multiple sequence alignment and evolutionary analysis of *PEBP* gene family

The 29 *PEBP* gene family sequences obtained in *B.napus* were completely aligned using MEGA 11.0 software [46]. First, MEGA 11.0 software was used to perform multiple sequence alignment of selected gene coding region sequences and translate them into amino acid sequences to construct an evolutionary tree through ClusterW. The Allgnment parameter was selected from the ClusterW options. Pairwise alignment was performed with gap opening penalty and gap extension penalty values of 15.00 and 6.66, respectively. Multiple Alignment was performed with a gap opening penalty and gap extension penalty of 15.00 and 6.66, respectively. Multiple sequences were aligned,and and the results were saved in MEGA format. A molecular evolutionary phylogenetic tree was constructed by the neighbor-joining (NJ) method, and the bootstrap parameter was 1000. The substitution type in the "substitution model" was "Nuckeotide," and the "Model/Method" as the "Tamura Nei model." Other parameters were defined as follows: Rates among sites in "Rates and patterns" as " Uniform rates, " " the Gap/Sending data treatment in Data subset to use" as "use all sites, " the ML heuristic method in "Tree input options" as "Nearest Highborn Interchange (NNI) ", and "the Initial tree for ML" as "Make initial tree automatically (Default NJ/BioNJ), " to build an evolutionary tree, and "Tree Style" to "Circle.". After preliminary construction of the evolutionary tree, the EvolView website (www.evolgenius.infi/evolview/#/terrview) was used for further beautification.

### Gene structure and motif prediction of *BnPEBP* genes

The annotation files of *PEBP* gene family members were downloaded from the BnTIR website (http://yanglab.hzau.edu.cn/). Gene structure was subsequently analysed, and a gene structure map was drawn by online Gene Structure Display Server (GSDS) analysis (http://gsds.gao-lab.org/) [47]. MEME software online (http://meme-suite.org/tools/meme) was used to identify the conserved motifs of the PEBP protein sequence in *B.napus* [48]. The number of motifs was set to 10, and the remaining parameters were set to default conditions. Finally, TBtools was used to visualize the gene evolutionary tree, gene structure, domains, and conserved motifs [49].

### Chromosomal distribution and homology analysis of *BnPEBP* genes

According to the information within the BnTIR database (http://yanglab.hzau.edu.cn/), the specific positions of the *PEBP* gene family members on the chromosomes of *B.napus* were analysed. Then, TBtools software was used to map these genes to individual chromosomes.

Gene duplication is considered one of the main drivers of the evolution of genomes and genetic systems [50]. It includes six different mechanisms (WGD, tandem, proximate, DNA-based transmission, retrotransmission, and separation). Among these patterns, fragments and tandem repeats are considered to be two main methods for the expansion of plant gene families [51]. Segmental duplication replicates multiple genes by genome doubling, usually on different chromosomes, and tandem duplications are characterized by regions of chromosomal recombination where multiple gene members are arranged in the same intergenic region or adjacent intergenic regions on the same chromosome [52, 53]. *B.napus* is an allotetraploid plant species formed by natural genome duplication. Many gene chromosomal segmental duplication events have occurred during the long-term evolutionary process. We are interested in segmented and tandem repeat events. FASTA genomic data files and GFF3 gene annotation files of *B.napus*, *B.rapa*, *B.oleracea,* and *A.thaliana* were downloaded from the Ensembl Plants website (http://plants.ensembl.org/index.html), TBtools was used to analyse the collinearity genes of the *PEBP* gene within *B.napus* species, between *B.napus* and *B.rapa*, *B.oleracea,* and *A.thaliana*, and visualize the results.

### Physicochemical properties and subcellular localization of *BnPEBP* genes

The physicochemical properties, including the number of amino acids, most abundant amino acids, molecular weight (MW), isoelectric point (pI), instability coefficient, hydrophobicity coefficient, grand average of hydropathy (GRAVY) value, and subcellular localization, of all *BnPEBP* proteins were predicted using the ExPASy website (http://web.expasy.org/protparam/) [54].

### Selection pressure analysis

In the process of evolutionary analysis, the Ka/Ks value is used to infer natural selection on genes and to determine the degree of environmental influence on homologous genes during the evolutionary process, as well as the direction of gene mutation. If Ka/Ks is greater than one, there is a positive selection effect; if Ka/Ks is less than one, there is neutral selection; and if Ka/Ks is one, there is purifying selectivition. The ratio of non-synonymous mutations (Ka) to synonymous mutations (Ks) was calculated by the Tbtools built-in Ka/Ks Calculator estimate the selection pressure on the 29 *PEBP* gene family members in *B.napus* over the course of evolution, with *A.thaliana PEBP* family genes as a reference.

### Analysis of cis-acting elements in the *BnPEBP* promoter

The 2000 bp sequence upstream of the *BnPEBP* genes was extracted using TBtools to analyses *cis-*acting elements, sequences were organized in one text file, and then PlantCARE (http://bioinformatics.psb.ugent.be/webtools/plantcare/html/) [55] was used to identify *cis*-acting elements. The files were upload, searches were performed in batches, and the final result was fed back as a TXT file. TBtools was then used to visualize the results.

### Interacting protein predictions

The STRING database contains a collection of protein interaction data from many species [56]. Sequences of the 29 *BnPEBP* genes orthologous to those in *A.thaliana* were input into the STRING database to identify the homologous *BnPEBP* genes in *B.napus*. The source genes were predicted via protein interaction networks, the prediction results were saved as a text file, and Cytoscape 3.7.2 was ultimately used to visualize the results [57].

### Tissue-specific expression analysis of *BnPEBP* family genes

To further analyse the expression of *BnPEBP* family genes in *B.napus* tissue, the expression data of 29 *BnPEBP* family genes in different tissues and at different times of *B.napus* cultivar "Zhongshuang 11 were downloaded from the BnTIR website (http://yanglab.hzau.edu.cn/). TBtools was used to draw a heatmap based on the log2-transformed expression data from each family member.

### qRT‒PCR analysis of *BnPEBP* family genes

Based on the expression analysis results of *PEBP* gene in different tissues of *B.napus*, we selected *B.napus* "Zhongshuang 9" as the experimental material and collected five tissues (root, stem, leaf, bud, and silique) of three plants with consistent growth at 10 days after flowering for a real-time fluorescence quantitative PCR assay. The qRT***‒***PCR quantitative experiment was completed with a kit from Shanghai Yeasen Biological Company. The first round of reverse transcription was performed with Hifair® III 1st Strand cDNA Synthesis SuperMix for qPCR® and QPCR SYBR Green Master Mix (Low Rox Plus). Primer 5.0 software was used to design specific primers for quantitative analysis (Supplementary Table S5), and Actin7 (GeneBank ID:GBEQ01027912.1) was used as an internal reference gene. Real-time PCR was performed with an AB-7500 fluorescence quantitative PCR apparatus, with three biological replicates and three technical replicates for each tissue, Relative expression of genes was quantified using the 2^−△△CT^ method to identify the expression patterns of the *BnPEBP* genes in different tissues of *B.napus.*

## Supplementary Information

**Additional file 1: Table S1.** The gene pairs and duplicated type of PEBP genes in B. napus.

**Additional file 2: Table S2.** The list of orthologous PEBP gene pairs between B. napus, B. oleracea, B. rapa, and A. thaliana.

**Additional file 3: Table S3.** Physicochemical properties and subcellular localization of PEBP gene family members in B.napus.

**Additional file 4: Table S4.** Ka/Ks values of BnPEBP genes between B.napus and A.thaliana.

**Additional file 5: Table S5.** Specific primers of BnPEBP genes for qRT-PCR.

## Data Availability

All data generated or analysed during this study are contained in the following persistent WEB links or access dataset. InterPro website: (https://www.ebi.ac.uk/interpro/) the *PEBP* family is included in the InterPro database under number ‘IPR008914’. HMMER website: https://www.ebi.ac.uk/Tools/hmmer/. Arabidopsis Information Resource website: https://www.arabidopsis.org/. The *PEBP* family member is included in the Arabidopsis database under number ‘*AT1G65480*, *AT4G20370*, *AT1G18100*, *AT5G03840*, *AT2G27550*, *AT5G62040*’. BRAD database (*B.napus* Zhongshuang11 genome, *B.rapa* Chiifu_V3.0 genome and *Brassica oleracea* JZS_V2.0 genome information): http://brassicadb.cn. Gene expression data were downloaded through the BnIR website: http://yanglab.hzau.edu.cn/, which integrates transcriptome data for all genes of Zhongshuang 11. Open the website and enter the gene name to download gene expression data without accession numbers. ExPASy online website: https://web.expasy.org/protparam/. Ensembl Plants website: http://plants.ensembl.org/index.html. PlantCARE website: http://bioinformatics.psb.ugent.be/webtools/plantcare/html/.
