# Supplementary material for: Identification, evolution and expression analyses of the whole genome-wide PEBP gene family in Brassica napus L
Source: BMC Genom Data. 2023 May 3;24:27. doi: 10.1186/s12863-023-01127-4 (PMC10155459; doi:10.1186/s12863-023-01127-4)
Supplement: Supplementary file 1 — Additional file 1: Table S1. The gene pairs and duplicated type of PEBP genes in B. napus. [file 12863_2023_1127_MOESM1_ESM.docx]

**Table S1. The gene pairs and duplicated type of *PEBP* genes in *B. napus***

| **Gene** | **Position start** | **Position end** | **Gene** | **Position start** | **Position end** | **Duplicated type** | **NO.GBS** |
| --- | --- | --- | --- | --- | --- | --- | --- |
| *BnaA02G0014100ZS* | 1037220 | 1038340 | *BnaA03G0012400ZS* | 588562 | 589627 | Segmental duplication | 255,255,204 |
| *BnaA02G0156900ZS* | 9104462 | 9107270 | *BnaA07G0282700ZS* | 26253195 | 26255955 | Segmental duplication | 228,26,28 |
| *BnaA02G0014100ZS* | 1037220 | 1038340 | *BnaA10G0288700ZS* | 25924219 | 25925284 | Segmental duplication | 255,255,204, |
| *BnaA02G0014100ZS* | 1037220 | 1038340 | *BnaC02G0013900ZS* | 1087793 | 1088870 | Segmental duplication | 255,255,204 |
| *BnaA02G0156900ZS* | 9104462 | 9107270 | *BnaC02G0200600ZS* | 16837203 | 16838959 | Segmental duplication | 228,26,28 |
| *BnaA02G0014100ZS* | 1037220 | 1038340 | *BnaC03G0016500ZS* | 785008 | 786061 | Segmental duplication | 255,255,204 |
| *BnaA02G0156900ZS* | 9104462 | 9107270 | *BnaC06G0323800ZS* | 42937446 | 42939746 | Segmental duplication | 228,26,28 |
| *BnaA02G0014100ZS* | 1037220 | 1038340 | *BnaC09G0608000ZS* | 67291091 | 67292162 | Segmental duplication | 255,255,204 |
| *BnaA03G0012400ZS* | 588562 | 589627 | *BnaA10G0288700ZS* | 25924219 | 25925284 | Segmental duplication | 0,102,102 |
| *BnaA03G0012400ZS* | 588562 | 589627 | *BnaC02G0013900ZS* | 1087793 | 1088870 | Segmental duplication | 0,102,102 |
| *BnaA03G0012400ZS* | 588562 | 589627 | *BnaC03G0016500ZS* | 785008 | 786061 | Segmental duplication | 0,102,102 |
| *BnaA03G0233400ZS* | 12179465 | 12181287 | *BnaC03G0275900ZS* | 17403007 | 17406731 | Segmental duplication | 0,153,153 |
| *BnaA03G0233400ZS* | 12179465 | 12181287 | *BnaC04G0478300ZS* | 60565511 | 60567293 | Segmental duplication | 0,153,153 |
| *BnaA03G0012400ZS* | 588562 | 589627 | *BnaC09G0608000ZS* | 67291091 | 67292162 | Segmental duplication | 0,102,102 |
| *BnaA04G0179000ZS* | 18389029 | 18389474 | *BnaC04G0478300ZS* | 60565511 | 60567293 | Segmental duplication | 141,211,199 |
| *BnaA04G0179000ZS* | 18389029 | 18389474 | *BnaC04G0205900ZS* | 20146840 | 20149549 | Segmental duplication | 14,211,199 |
| *BnaA06G0123900ZS* | 7315661 | 7317429 | *BnaA09G0615100ZS* | 60355245 | 60356970 | Segmental duplication | 204,0,204 |
| *BnaA06G0273500ZS* | 37354175 | 37355019 | *BnaC03G0559000ZS* | 42600265 | 42601116 | Segmental duplication | 153,204,255 |
| *BnaA06G0123900ZS* | 7315661 | 7317429 | *BnaC05G0152000ZS* | 9745846 | 9747465 | Segmental duplication | 204,0,204 |
| *BnaA07G0282700ZS* | 26253195 | 26255955 | *BnaC02G0200600ZS* | 16837203 | 16838959 | Segmental duplication | 27,158,119 |
| *BnaA07G0365100ZS* | 30986418 | 30988169 | *BnaC02G0302200ZS* | 29002223 | 29003948 | Segmental duplication | 31,120,180 |
| *BnaA07G0155500ZS* | 18656500 | 18658099 | *BnaC04G0478300ZS* | 60565511 | 60567293 | Segmental duplication | 253,194,134 |
| *BnaA07G0155500ZS* | 18656500 | 18658099 | *BnaC04G0205900ZS* | 20146840 | 20149549 | Segmental duplication | 253,194,134 |
| *BnaA07G0282700ZS* | 26253195 | 26255955 | *BnaC06G0323800ZS* | 42937446 | 42939746 | Segmental duplication | 27,158,119 |
| *BnaA07G0365100ZS* | 30986418 | 30988169 | *BnaC06G0428800ZS* | 50699507 | 50700480 | Segmental duplication | 31,120,180 |
| *BnaA09G0615100ZS* | 60355245 | 60356970 | *BnaC05G0152000ZS* | 9745846 | 9747465 | Segmental duplication | 252,205,229 |
| *BnaA09G0615100ZS* | 60355245 | 60356970 | *BnaC08G0470600ZS* | 49930224 | 49931954 | Segmental duplication | 252,205,229 |
| *BnaA10G0288700ZS* | 25924219 | 25925284 | *BnaC03G0016500ZS* | 785008 | 786061 | Segmental duplication | 31,120,180 |
| *BnaA10G0288700ZS* | 25924219 | 25925284 | *BnaC09G0608000ZS* | 67291091 | 67292162 | Segmental duplication | 31,120,180 |
| *BnaC02G0013900ZS* | 1087793 | 1088870 | *BnaC03G0016500ZS* | 785008 | 786061 | tandem duplication | 190,186,218 |
| *BnaC02G0200600ZS* | 16837203 | 16838959 | *BnaC06G0323800ZS* | 42937446 | 42939746 | Segmental duplication | 255,255,51 |
| *BnaC02G0302200ZS* | 29002223 | 29003948 | *BnaC06G0428800ZS* | 50699507 | 50700480 | Segmental duplication | 102,102,102 |
| *BnaC02G0013900ZS* | 1087793 | 1088870 | *BnaC09G0608000ZS* | 67291091 | 67292162 | Segmental duplication | 190,186,218 |
| *BnaC03G0016500ZS* | 785008 | 786061 | *BnaC09G0608000ZS* | 67291091 | 67292162 | Segmental duplication | 231,41,138 |
| *BnaC05G0152000ZS* | 9745846 | 9747465 | *BnaC08G0470600ZS* | 49930224 | 49931954 | Segmental duplication | 0,255,51 |
