# Supplementary material for: Identification, evolution and expression analyses of the whole genome-wide PEBP gene family in Brassica napus L
Source: BMC Genom Data. 2023 May 3;24:27. doi: 10.1186/s12863-023-01127-4 (PMC10155459; doi:10.1186/s12863-023-01127-4)
Supplement: Supplementary file 2 — Additional file 2: Table S2. The list of orthologous PEBP gene pairs between B. napus, B. oleracea, B. rapa, and A. thaliana. [file 12863_2023_1127_MOESM2_ESM.docx]

**Table S2. The list of orthologous *PEBP* gene pairs between *B. napus*, *B. oleracea*, *B. rapa, and A. thaliana*.**

| ***B. napus vs B. oleracea*** | | ***B. napus vs B. rapa*** | | ***B. napus vs A. Ghaliana*** | |
| --- | --- | --- | --- | --- | --- |
| *BnaA02G0014100ZS* | *Bo2g005260.1* | *BnaA02G0014100ZS* | *BrA02p001260.1* | *BnaA06G0123900ZS* | *AT1G18100.1* |
| *BnaA03G0012400ZS* | *Bo2g005260.1* | *BnaA03G0012400ZS* | *BrA02p001260.1* | *BnaA09G0615100ZS* | *AT1G18100.1* |
| *BnaA10G0288700ZS* | *Bo2g005260.1* | *BnaA10G0288700ZS* | *BrA02p001260.1* | *BnaC05G0152000ZS* | *AT1G18100.1* |
| *BnaC02G0013900ZS* | *Bo2g005260.1* | *BnaC02G0013900ZS* | *BrA02p001260.1* | *BnaC08G0470600ZS* | *AT1G18100.1* |
| *BnaC03G0016500ZS* | *Bo2g005260.1* | *BnaC03G0016500ZS* | *BrA02p001260.1* | *BnaA03G0233400ZS* | *AT2G27550.1* |
| *BnaC09G0608000ZS* | *Bo2g005260.1* | *BnaC09G0608000ZS* | *BrA02p001260.1* | *BnaA04G0179000ZS* | *AT2G27550.1* |
| *BnaA03G0233400ZS* | *Bo3g040460.1* | *BnaA02G0014100ZS* | *BrA03p001500.1* | *BnaA07G0155500ZS* | *AT2G27550.1* |
| *BnaA04G0179000ZS* | *Bo3g040460.1* | *BnaA03G0012400ZS* | *BrA03p001500.1* | *BnaC03G0275900ZS* | *AT2G27550.1* |
| *BnaA06G0273500ZS* | *Bo3g108060.1* | *BnaA03G0233400ZS* | *BrA03p027680.1* | *BnaC04G0478300ZS* | *AT2G27550.1* |
| *BnaA07G0155500ZS* | *Bo3g040460.1* | *BnaA04G0179000ZS* | *BrA03p027680.1* | *BnaC04G0205900ZS* | *AT2G27550.1* |
| *BnaC03G0275900ZS* | *Bo3g040460.1* | *BnaA07G0155500ZS* | *BrA03p027680.1* | *BnaA02G0014100ZS* | *AT5G03840.1* |
| *BnaC03G0559000ZS* | *Bo3g108060.1* | *BnaA10G0288700ZS* | *BrA03p001500.1* | *BnaA03G0012400ZS* | *AT5G03840.1* |
| *BnaC04G0205900ZS* | *Bo3g040460.1* | *BnaC02G0013900ZS* | *BrA03p001500.1* | *BnaA06G0273500ZS* | *AT5G62040.1* |
| *BnaC04G0478300ZS* | *Bo3g040460.1* | *BnaC03G0016500ZS* | *BrA03p001500.1* | *BnaA10G0288700ZS* | *AT5G03840.1* |
| *BnaA03G0233400ZS* | *Bo4g074330.1* | *BnaC03G0275900ZS* | *BrA03p027680.1* | *BnaC02G0013900ZS* | *AT5G03840.1* |
| *BnaA03G0233400ZS* | *Bo4g164740.1* | *BnaC04G0205900ZS* | *BrA03p027680.1* | *BnaC03G0016500ZS* | *AT5G03840.1* |
| *BnaA04G0179000ZS* | *Bo4g164740.1* | *BnaC04G0478300ZS* | *BrA03p027680.1* | *BnaC03G0559000ZS* | *AT5G62040.1* |
| *BnaA04G0179000ZS* | *Bo4g074330.1* | *BnaC09G0608000ZS* | *BrA03p001500.1* | *BnaC09G0608000ZS* | *AT5G03840.1* |
| *BnaA07G0155500ZS* | *Bo4g164740.1* | *BnaA03G0233400ZS* | *BrA04p026440.1* |  |  |
| *BnaA07G0155500ZS* | *Bo4g074330.1* | *BnaA04G0179000ZS* | *BrA04p026440.1* |  |  |
| *BnaC03G0275900ZS* | *Bo4g074330.1* | *BnaA07G0155500ZS* | *BrA04p026440.1* |  |  |
| *BnaC03G0275900ZS* | *Bo4g164740.1* | *BnaC03G0275900ZS* | *BrA04p026440.1* |  |  |
| *BnaC04G0478300ZS* | *Bo4g164740.1* | *BnaC04G0478300ZS* | *BrA04p026440.1* |  |  |
| *BnaC04G0205900ZS* | *Bo4g074330.1* | *BnaC04G0205900ZS* | *BrA04p026440.1* |  |  |
| *BnaC04G0205900ZS* | *Bo4g164740.1* | *BnaA06G0273500ZS* | *BrA06p035460.1* |  |  |
| *BnaC04G0478300ZS* | *Bo4g074330.1* | *BnaA06G0123900ZS* | *BrA06p015480.1* |  |  |
| *BnaA06G0123900ZS* | *Bo5g025100.1* | *BnaA09G0615100ZS* | *BrA06p015480.1* |  |  |
| *BnaA09G0615100ZS* | *Bo5g025100.1* | *BnaC03G0559000ZS* | *BrA06p035460.1* |  |  |
| *BnaC05G0152000ZS* | *Bo5g025100.1* | *BnaC05G0152000ZS* | *BrA06p015480.1* |  |  |
| *BnaC08G0470600ZS* | *Bo5g025100.1* | *BnaC08G0470600ZS* | *BrA06p015480.1* |  |  |
| *BnaA07G0365100ZS* | *Bo6g120900.1* | *BnaA03G0233400ZS* | *BrA07p023970.1* |  |  |
| *BnaC02G0302200ZS* | *Bo6g120900.1* | *BnaA04G0179000ZS* | *BrA07p023970.1* |  |  |
| *BnaC06G0428800ZS* | *Bo6g120900.1* | *BnaA07G0155500ZS* | *BrA07p023970.1* |  |  |
| *BnaA06G0123900ZS* | *Bo8g104520.1* | *BnaA07G0365100ZS* | *BrA07p049550.1* |  |  |
| *BnaA09G0615100ZS* | *Bo8g104520.1* | *BnaC02G0302200ZS* | *BrA07p049550.1* |  |  |
| *BnaC05G0152000ZS* | *Bo8g104520.1* | *BnaC03G0275900ZS* | *BrA07p023970.1* |  |  |
| *BnaC08G0470600ZS* | *Bo8g104520.1* | *BnaC04G0478300ZS* | *BrA07p023970.1* |  |  |
| *BnaA02G0014100ZS* | *Bo9g181670.1* | *BnaC04G0205900ZS* | *BrA07p023970.1* |  |  |
| *BnaA03G0012400ZS* | *Bo9g181670.1* | *BnaC06G0428800ZS* | *BrA07p049550.1* |  |  |
| *BnaA10G0288700ZS* | *Bo9g181670.1* | *BnaA06G0123900ZS* | *BrA09p073710.1* |  |  |
| *BnaC02G0013900ZS* | *Bo9g181670.1* | *BnaA09G0615100ZS* | *BrA09p073710.1* |  |  |
| *BnaC03G0016500ZS* | *Bo9g181670.1* | *BnaC05G0152000ZS* | *BrA09p073710.1* |  |  |
| *BnaC09G0608000ZS* | *Bo9g181670.1* | *BnaC08G0470600ZS* | *BrA09p073710.1* |  |  |
|  |  | *BnaA02G0014100ZS* | *BrA10p039580.1* |  |  |
|  |  | *BnaA03G0012400ZS* | *BrA10p039580.1* |  |  |
|  |  | *BnaA10G0288700ZS* | *BrA10p039580.1* |  |  |
|  |  | *BnaC02G0013900ZS* | *BrA10p039580.1* |  |  |
|  |  | *BnaC03G0016500ZS* | *BrA10p039580.1* |  |  |
|  |  | *BnaC09G0608000ZS* | *BrA10p039580.1* |  |  |
