# Supplementary material for: Identification, evolution and expression analyses of the whole genome-wide PEBP gene family in Brassica napus L
Source: BMC Genom Data. 2023 May 3;24:27. doi: 10.1186/s12863-023-01127-4 (PMC10155459; doi:10.1186/s12863-023-01127-4)
Supplement: Supplementary file 3 — Additional file 3: Table S3. Physicochemical properties and subcellular localization of PEBP gene family members in B.napus. [file 12863_2023_1127_MOESM3_ESM.docx]

**Table S3.** **Physicochemical properties and subcellular localization of *PEBP* gene family members in *B.napus*.**

| ***B. napu* ID** | **Number of amino acids** | **Acid (%)** | **Molecular weight** | **Theoretical pI** | **Instability index** | **Aliphatic index** | **GRAVY** | **Subcellular localization** |
| --- | --- | --- | --- | --- | --- | --- | --- | --- |
| *BnaFT-A02* | *175* | *Val (V) 10.9%* | *19810.4* | *7.75* | *46.66* | *87.26* | *-0.338* | *Cytoplasmic* |
| *BnaFT-A07* | *175* | *Val (V)10.3%;*  *Arg (R)10.3%;* | *19772.4* | *7.82* | *38.39* | *88.4* | *-0.338* | *Cytoplasmic* |
| *BnaFT-A07* | *176* | *Val (V) 9.7%;*  *Leu (L) 9.7%;* | *19945.7* | *7.72* | *39.17* | *84.6* | *-0.293* | *Extracellular* |
| *BnaFT-C02* | *175* | *Val (V)10.9%* | *19810.4* | *7.75* | *46.66* | *87.26* | *-0.342* | *Cytoplasmic* |
| *BnaFT-C04* | *175* | *Leu (L)10.9%* | *19860.6* | *6.9* | *36.16* | *85.66* | *-0.271* | *Cytoplasmic* |
| *BnaFT-C06* | *175* | *Arg (R) 10.3%* | *19773.4* | *7.75* | *35.23* | *87.31* | *-0.349* | *Cytoplasmic* |
| *BnaTSF-C02* | *202* | *Leu (L)11.4%* | *22959.5* | *8.63* | *51.09* | *93.02* | *-0.076* | *Cytoplasmic* |
| *BnaTSF-C06* | *142* | *Leu (L)10.6%* | *16095.7* | *8.46* | *45.03* | *100.7* | *-0.186* | *Cytoplasmic* |
| *BnaMFT-A06* | *173* | *Pro (P)12.7%* | *18917* | *7.87* | *42.91* | *85.55* | *-0.112* | *Periplasmic* |
| *BnaMFT-A09* | *173* | *Pro (P)12.1%* | *19070* | *8.79* | *48.44* | *79.36* | *-0.174* | *Periplasmic* |
| *BnaMFT-C05* | *174* | *Val (V)11.5%;*  *Pro(P) 11.5%* | *19081.1* | *7.88* | *49.73* | *86.72* | *-0.099* | *Periplasmic* |
| *BnaMFT-C08* | *173* | *Pro (P) 12.1%* | *19043* | *8.79* | *45.9* | *79.36* | *-0.158* | *Periplasmic* |
| *BnaTFL1-A02* | *179* | *Val (V) 10.6%* | *20435.5* | *8.79* | *48.89* | *84.8* | *-0.191* | *Cytoplasmic* |
| *BnaTFL1-A03* | *177* | *Val (V) 10.2%* | *20083.2* | *9.56* | *49.01* | *86.89* | *-0.173* | *Cytoplasmic* |
| *BnaTFL1-A10* | *178* | *Val (V) 10.7%* | *20401.5* | *9.51* | *49.66* | *85.28* | *-0.317* | *Cytoplasmic* |
| *BnaTFL1-C02* | *179* | *Val (V) 10.6%* | *20484.6* | *8.76* | *46.44* | *84.8* | *-0.179* | *Cytoplasmic* |
| *BnaTFL1-C03* | *174* | *Val (V) 10.3%* | *19847.9* | *9.56* | *50.55* | *85.57* | *-0.154* | *Cytoplasmic* |
| *BnaTFL1-C09* | *178* | *Val (V) 10.7%* | *20401.5* | *9.51* | *49.66* | *85.28* | *-0.317* | *Cytoplasmic* |
| *BnaTFL1-A03* | *175* | *Val (V) 12.0%* | *19819.7* | *7.01* | *29.64* | *78.91* | *-0.232* | *Cytoplasmic* |
| *BnaATC-A04* | *86* | *Val (V) 14.0%* | *9613.17* | *7.74* | *26.9* | *84.77* | *0.034* | *Cytoplasmic* |
| *BnaATC-A07* | *175* | *Val (V) 12.0%* | *19775.6* | *7.01* | *35.55* | *77.83* | *-0.236* | *Cytoplasmic* |
| *BnaATC-C03* | *175* | *Val (V) 12.0%* | *19750.7* | *7.79* | *28.74* | *82.29* | *-0.182* | *Cytoplasmic* |
| *BnaATC-C04* | *76* | *Val (V) 14.5%* | *8409.77* | *6.38* | *39.1* | *84.47* | *0.086* | *Periplasmic* |
| *BnaATC-C04* | *175* | *Val (V) 12.0%* | *19888.8* | *7.76* | *36.97* | *76.69* | *-0.273* | *Cytoplasmic* |
| *BnaATC-0027* | *181* | *Val (V) 11.0%* | *20598.5* | *8.47* | *33.23* | *75.8* | *-0.298* | *Cytoplasmic* |
| *BnaATC-0105* | *175* | *Val (V) 12.0%* | *19775.6* | *7.01* | *35.55* | *77.83* | *-0.236* | *Cytoplasmic* |
| *BnaATC-0139* | *175* | *Val (V) 12.1%* | *19775.6* | *7.01* | *34.99* | *77.83* | *-0.236* | *Cytoplasmic* |
| *BnaBFT-A06* | *177* | *Arg (R) 10.7%* | *20146.1* | *9.51* | *51.58* | *75.42* | *-0.237* | *Cytoplasmic* |
| *BnaBFT-C03* | *177* | *Arg (R) 10.7%* | *20132.1* | *9.51* | *51.58* | *74.86* | *-0.235* | *Cytoplasmic* |
