# Supplementary material for: Identification, evolution and expression analyses of the whole genome-wide PEBP gene family in Brassica napus L
Source: BMC Genom Data. 2023 May 3;24:27. doi: 10.1186/s12863-023-01127-4 (PMC10155459; doi:10.1186/s12863-023-01127-4)
Supplement: Supplementary file 4 — Additional file 4: Table S4. Ka/Ks values of BnPEBP genes between B.napus and A.thaliana. [file 12863_2023_1127_MOESM4_ESM.docx]

**Table S4.** **Ka/Ks values of *BnPEBP* genes between *B.napus* and *A.thaliana.***

| ***Gene name in A. thaliana*** | ***Gene name in B. napus*** | ***Ka*** | ***Ks*** | ***Ka/Ks*** |
| --- | --- | --- | --- | --- |
| *AtFT* | *BnFT-A02* | *0.357196399* | *1.458844* | *0.218902* |
| *AtFT* | *BnFT-A07-1* | *0.376866512* | *2.057615* | *0.212526* |
| *AtFT* | *BnFT-A07-2* | *0.429324752* | *1.982417* | *0.229618* |
| *AtFT* | *BnFT-C02* | *0.394072304* | *1.820495* | *0.229849* |
| *AtFT* | *BnFT-C04* | *0.410821762* | *2.309814* | *0.198682* |
| *AtFT* | *BnFT-C06* | *0.430750267* | *2.342328* | *0.186762* |
| *AtTSF* | *BnTSF-C02* | *0.468931855* | *2.563451* | *0.200702* |
| *AtTSF* | *BnTSF-C06* | *0.6972822* | *2.295324* | *0.31564* |
| *AtMFT* | *BnMFT-A06* | *0.400690945* | *2.133973* | *0.201752* |
| *AtMFT* | *BnMFT-A09* | *0.400002227* | *2.396206* | *0.121076* |
| *AtMFT* | *BnMFT-C05* | *0.445808067* | *2.285331* | *0.1804* |
| *AtMFT* | *BnMFT-C08* | *0.43660519* | *3.299905* | *0.141075* |
| *AtATC* | *BnATC-A03* | *0.139546128* | *1.252361* | *0.12629* |
| *AtATC* | *BnATC-A04* | *0.185387726* | *0.584192* | *0.238098* |
| *AtATC* | *BnATC-A07* | *0.155062432* | *1.27188* | *0.224371* |
| *AtATC* | *BnATC-C03* | *0.175398277* | *1.495517* | *0.132125* |
| *AtATC* | *BnATC-C04-1* | *0.265606689* | *1.232802* | *0.33837* |
| *AtATC* | *BnATC-C04-2* | *0.342756521* | *1.891904* | *0.201809* |
| *AtATC* | *BnATC-027* | *0.218320901* | *1.746224* | *0.280674* |
| *AtATC* | *BnATC-105* | *0.239835483* | *1.596848* | *0.210352* |
| *AtATC* | *BnATC-139* | *0.255081467* | *2.436836* | *0.116474* |
| *AtTFL1* | *BnTFL1-A03* | *0.137318345* | *0.498558* | *0.367131* |
| *AtTFL1* | *BnTFL1-A10* | *0.238662543* | *1.634074* | *0.235288* |
| *AtTFL1* | *BnTFL1-C02* | *0.197260224* | *0.718426* | *0.297852* |
| *AtTFL1* | *BnTFL1-C03* | *0.191080371* | *0.950353* | *0.30009* |
| *AtTFL1* | *BnTFL1-C09* | *0.298981798* | *1.199302* | *0.249392* |
| *AtBTF* | *BnBTF-A06* | *0.002516781* | *0.022903* | *0.109891* |
