# Supplementary material for: Identification, evolution and expression analyses of the whole genome-wide PEBP gene family in Brassica napus L
Source: BMC Genom Data. 2023 May 3;24:27. doi: 10.1186/s12863-023-01127-4 (PMC10155459; doi:10.1186/s12863-023-01127-4)
Supplement: Supplementary file 5 — Additional file 5: Table S5. Specific primers of BnPEBP genes for qRT-PCR. [file 12863_2023_1127_MOESM5_ESM.docx]

**Table S5. Specific primers of *BnPEBP* genes for qRT-PCR**

| **Gene name** | **Sequence(5’-3’)** |
| --- | --- |
| *BnaA02G0156900ZS* | TCATCGTATCGTGCTCGTATTG |
|  | GGGAAGGCCGAGATTGTATAG |
| *BnaA07G0282700ZS* | ATCGTCTCGTGCTGGTATTG |
|  | GGGAAGGCCGAGATTGTATAG |
| *BnaA07G0365100ZS* | GGTTGAGATTGGAGGAGATGAC |
|  | TCACCAACCAATGGAGGTATTC |
| *BnaC02G0200600ZS* | TCATCGTATCGTGCTGGTATTG |
|  | GGGAAGGCCGAGATTGTATAG |
| *BnaC04G0181400ZS* | GGCCAGAGACAGGTTACTAATG |
|  | TCCACCATAACCAAAGTGTAGAA |
| *BnaC06G0323800ZS* | GGGTTACATACGGCCAAAGA |
|  | CCTTAGGTCTTCTCCACCAATC |
| *BnaC02G0302200ZS* | TGAGAACCCATGTCCCAATTC |
|  | CGCCACTCAGGTGCATAAA |
| *BnaC06G0428800ZS* | CGGTTGAGATTGGAGGAGATG |
|  | ATGGAGGTATTCTCGGAGGT |
| *BnaA06G0123900ZS* | CTAACGGCTGCGAGATCAAA |
|  | CAGTCATCACGAGGGTGTAAAG |
| *BnaA09G0615100ZS* | GTTGGAAGAGTGATCGGAGATG |
|  | ATCTCGCAGCCGTTAGTTATG |
| *BnaC05G0152000ZS* | CTAACGGCTGCGAGATCAAA |
|  | CAGTCATCACGAGGGTGTAAAG |
| *BnaC08G0470600ZS* | GTTGGAAGAGTGATCGGAGATG |
|  | TGATCTCACAGCCGTTAGTTATG |
| *BnaA03G0233400ZS* | TGGACCTAGCGATCCTTATCT |
|  | GGCATCTCGTACCCTACTATTTC |
| *BnaA04G0179000ZS* | GATATCGTCAGACCCGCTTATG |
|  | TAGACTTGCTTGTCGCAGTTAT |
| *BnaA07G0155500ZS* | GATCCTTATCTCAGGGAGCATTT |
|  | GGCATCTCGTACCCTACTATTTC |
| *BnaC03G0275900ZS* | CTGGACCTAGTGACCCTTATCT |
|  | GGCATCTCGTACGCTACTATTT |
| *BnaC04G0205900ZS* | GACCCACTTATGGTTGGAAGAG |
|  | TGGCCATTGTAGACTTGCTTAT |
| *BnaC04G0478300ZS* | TCCTGGACCTAGTGATCCTTAT |
|  | GGCATCTCGTACCCTACTATTTC |
| *Bnascaffold0027G0054400ZS* | GACCCACTTATGGTTGGAAGAG |
|  | GTAGACTTGCTTGTCGCAGTTA |
| *Bnascaffold0105G0000300ZS* | GATCCTTATCTCAGGGAGCATTT |
|  | GGCATCTCGTACCCTACTATTTC |
| *BnaA02G0014100ZS* | CATCCCCGGTACAACAGATGCA |
|  | ACTTATCTCTCGAAGGAATGTT |
| *BnaA03G0012400ZS* | GAGCACCTGCATTGGATAGT |
|  | CACGAACCTGTGTATCCCTATG |
| *BnaA10G0288700ZS* | GCAGAAGCAAAGACGTGTTAAG |
|  | GACAGGGAGACCAAGATCATTC |
| *BnaC02G0013900ZS* | GATAGTGGGAAGAGTGGTAGGA |
|  | CTAGGCTTTGAGGAGACAAAGG |
| *BnaC03G0016500ZS* | ATGGGAGAAAGAGTGATAGAGC |
|  | CTCATGGCTGTTGGAGACTTGC |
| *BnaC09G0608000ZS* | GCAGAAGCAAAGACGTGTTAAG |
|  | GACAGGGAGACCAAGATCATTC |
| *BnaA06G0273500ZS* | CCGAGTCCTAGTAATCCCTACA |
|  | TCGTATCTCACCATCTCTCTCC |
| *BnaC03G0559000ZS* | AGTGGGAAGAGTGATAGGAGAT |
|  | GCCTTGAGAGGAGAACAGAAG |
| *q-actin73* | TGTGCTTGACTCTGGTGATGGT |
|  | GACGGAGGATAGCGTGAGGAAG |
